# Metasurface higher-order poincaré sphere polarization detection clock

**DOI:** 10.1038/s41377-024-01738-1

**Published:** 2025-01-26

**Authors:** Hui Yang, Kai Ou, Qiang Liu, Meiyu Peng, Zhenwei Xie, Yuting Jiang, Honghui Jia, Xinbin Cheng, Hui Jing, Yueqiang Hu, Huigao Duan

**Affiliations:** 1 National Research Center for High-Efficiency Grinding, College of Mechanical and Vehicle Engineering, Hunan University, 410082 Changsha, China; 2 School of Physics and Electronics, Hunan Normal University, 410081 Changsha, China; 3 Institute of Precision Optical Engineering, School of Physics Science and Engineering, Tongji University, 200092 Shanghai, China; 4 Nanophotonics Research Center, Shenzhen Key Laboratory of Micro-scale Optical Information Technology, Institute of Microscale Optoelectronics, Shenzhen University, 518060 Shenzhen, Guangdong China; 5 Greater Bay Area Institute for Innovation, Hunan University, 511300 Guangzhou, Guangdong Province China

**Keywords:** Nanophotonics and plasmonics, Nanophotonics and plasmonics

## Abstract

Accurately and swiftly characterizing the state of polarization (SoP) of complex structured light is crucial in the realms of classical and quantum optics. Conventional strategies for detecting SoP, which typically involves a sequence of cascaded optical elements, are bulky, complex, and run counter to miniaturization and integration. While metasurface-enabled polarimetry has emerged to overcome these limitations, its functionality predominantly remains confined to identifying SoP within the standard Poincaré sphere framework. The comprehensive detection of SoP on the higher-order Poincaré sphere (HOPS), however, continues to be a huge challenge. Here, we propose a general polarization metrology method capable of fully detecting SoP on any HOPS through a single measurement. The underlying mechanism relies on transforming the optical singularities and Stokes parameters into visualized intensity patterns, facilitating the extraction of all parameters that fully determine a SoP. We actualize this concept through a novel meta-device known as the metasurface photonics polarization clock, which offers an intuitive display of SoP using four distinct pointers. As a proof of concept, we theoretically and experimentally demonstrate fully resolving SoPs on the 0th, 1st, and 2nd HOPSs. Our implementation opens up a new pathway towards real-time polarimetry of arbitrary beams featuring miniaturized size, a simple detection process, and a direct readout mechanism, promising significant advancements in fields reliant on polarization.

## Introduction

Polarization, which is one of the fundamental characteristics of electromagnetic wave, characterizes the vector nature of the oscillating electric field. In order to fully describe the state of polarization (SoP), a geometric representation called the standard Poincaré sphere (PS) is proposed in 1892^1^. This representation maps all fundamental SoPs—circular, linear, and elliptical—onto the PS surface using Stokes parameters. Nevertheless, the standard PS only applies to homogeneous plane wave solutions derived from Maxwell’s vector wave equation that possess homogeneous polarization distribution. For higher-order solutions involving electromagnetic waves with inhomogeneous polarization distribution, a generalized geometric representation known as the higher-order Poincaré sphere (HOPS) has been introduced^2–4^. The HOPS incorporates both spin angular momentum (SAM) and orbital angular momentum (OAM), describing not only phase but also polarization evolution and providing a comprehensive representation for arbitrary space-variant polarization fields^3,5,6^. Similarly, the space-variant polarization fields can be mapped onto the HOPS surface using higher-order Stokes parameters. Notably, the standard PS is a subset of the HOPS, establishing the HOPS as a comprehensive framework for representing polarization states.

Light beam with spatially homogeneous or inhomogeneous SoP distribution plays a pivotal role in dictating light-matter interactions, underpinning numerous optical technologies. The vector beams with spatially inhomogeneous SoP distributions have garnered significant attention due to their potential applications across diverse fields^7,8^, such as optical communication, display technology, and encryption methods, particle manipulation techniques, high-dimensional quantum information processing. For this, the generation of vector beams has been explored using various platforms, including free space^5,6^, fiber end face^9^, on-chip platform^10^, and direct laser emission^3^. It is crucial to address the challenge of generating and detecting vector beams as a primary step towards realizing their practical applications. Consequently, precise identification of SoPs that denoted by points on the HOPS, is essential in numerous domains such as polarization imaging^11^, remote sensing^12^, material analysis^13^, optical trapping^14,15^, high-dimensional quantum technology^16,17^, optical data storage and communication^9,18–20^. However, direct measurements of SoPs are challenging due to phase information loss in conventional intensity-based detection schemes. To address this issue, the conventional wisdom of accurately determining the SoPs relies on implementing a series of intensity measurements by spatial or temporal division of input light waves^21^. Nevertheless, these processes introduce a set of optical elements (e.g., wave-plates and polarizers) along with intricate measurement protocols, leading to bulky setups and complicate measuring procedure that hinder the development of compact and integrated polarization detection devices.

Metasurfaces, artificial two-dimensional electromagnetic materials that go far beyond the physical limit of natural materials, provide a versatile platform for manipulating the properties of light in an almost unrestricted manner^22–25^. So far, all kinds of metasurface-enabled ultrathin functional meta-devices have been demonstrated, such as metalenses^26–29^, spectrometers^30^, meta-holograms^24,31–34^, and structured light generators^6,20,35^. Additionally, there has been significant interest in metasurface-enabled polarization detecting devices which can partially or fully resolve the polarization characteristics of incident light^36–45^. Initial attempt has been devoted to resolving photon spin via the elaborately designed on-chip silicon micro-disk or the chirality-coded meta-aperture arrays^46,47^. Subsequent studies focus on fully retrieved the Stokes parameters of beams on standard PS through polarization-dependent meta-gratings^36,48^, metalens^38,49^, meta-hologram^40^, among others. These meta-devices not only streamline the conventional process of polarization detection, but also align more effectively with the current trend of integrating photonic devices due to their subwavelength dimensions. Despite the exciting results, these compact polarimeters have been constrained to resolving SoPs on standard PS. For structured vector beams on the HOPS, some pioneering studies have managed to fully detect optical singularities, namely, the spin and angular momentum values in free space or on-chip^37,43,50–52^. Nevertheless, these approaches are limited in their inability to retrieve the Stokes parameters and can only determine the HOPS order without accurately pinpointing the specific surface location. Recently, fully and accurately determine the SoPs on the HOPS have been demonstrated by combining several polarization-dependent dielectric metalenses to form the Hartmann–Shack array^39,53^. Furthermore, a non-interleaved chiral metasurface incorporated with a convolutional neural network has been utilized for polarimetry with advantage of high-spatial-resolution^54^. However, this pixel-by-pixel polarization detection method necessitates complex comparative analyses with standard vector beams, resulting in relatively large-scale meta-devices. In general, a metrology method for fully detecting beams on the HOPS with a miniaturized size, simple detection process, and a direct readout mechanism is highly desired.

In this work, we overcome the restrictions of contemporary polarimeters and propose a metasurface photonics polarization clock (MPPC) that enables fully characterization of beams on any HOPS via a single measurement. The underlying mechanism relies on transforming optical singularities and Stokes parameters into specific intensity distributions on a transverse plane. By analyzing the characteristics (e.g. location and intensity strength) of the generated intensity distributions, we can obtain the required four parameters ( | *m*, *n*, 2*ψ, 2χ* > ) that fully characterize the polarization of an incident beam. The MPPC ingeniously visualizes the SoP (represented by these four parameters) with four pointers, akin to how a conventional clock displays time with three pointers. As a proof of concept, we theoretically and experimentally demonstrate fully resolving the SoPs on the 0th, 1st, and 2nd HOPSs. Moreover, fully resolving the SoPs on the hybrid-order HOPS_2,−1_ and 5th order HOPS are theoretically demonstrate as well. The designed MPPC possesses advantages of miniaturized size, simple detection process and direct readout mechanism, holding important applications in various fields such as remote sensing, optical trapping and high-dimensional quantum technology.

## Results

### Comparison of polarization detecting scheme between the conventional setup and the MPPC

To fully characterize the SoP of a monochromatic light beam, the HOPS representation is adopted and an arbitrary polarized beam located on the HOPS can be described as

1 $$|P\rangle =\,\cos (\chi ){e}^{i\varPsi }|{R}_{m}\rangle +\,\sin (\chi ){e}^{-i\varPsi }|{L}_{n}\rangle$$

where |${R}_{m}$ > $={e}^{im\varphi }$ | *R*> and |${L}_{n}$ > $={e}^{in\varphi }$ | *L*> are the two circularly polarized (CP)vortex bases. *m* and *n* denote the topological charges of the two CP vortex beams. The ellipticity and azimuth angle are respectively represented as *χ* and *ψ*, which can be derived as

2 $$2\chi =\pi /2-\arcsin ({S}_{3}^{m,n})$$

3 $$2\psi =\arctan ({S}_{2}^{m,n}/{S}_{1}^{m,n})$$

where ${S}_{i}^{m,n}$, *i* = 1, 2, 3 denote the higher-order Stokes parameters (see section 1 of the Supplementary file). Hence, an arbitrary beam with space-variant SoP can be effectively mapped to the surface of the HOPS using spherical coordinates (2*ψ, 2χ*), employing higher-order Stokes parameters. It is worth noting that when *m* = *n* = 0, the HOPS degrades into the standard PS, and correspondingly, the higher-order Stokes parameters also degrades into the standard ones. Consequently, throughout this text, we treat the standard PS as a distinctive case of the HOPS. According to Eqs. (2) and (3), it becomes apparent that in order to fully determine the SoP of an incident beam on the HOPS, a set of four parameters |*m*, *n*, 2*ψ, 2χ*> is required.

**Fig. 1:**
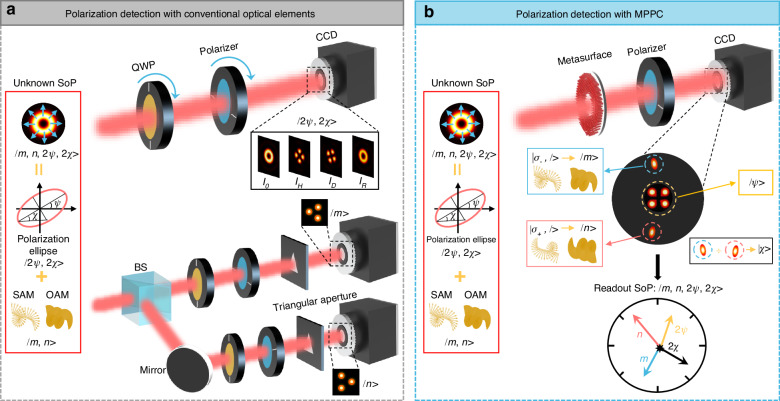
Comparison between the polarization detection processes of the conventional configuration and the proposed MPPC. **a** The traditional polarization detection system is schematically depicted, consisting of a series of optical elements such as polarizer, quarter-waveplate, mirrors and CCD. Two sets of experimental setups are needed to detect the Stokes parameters |2*ψ*, 2*χ*> and optical singularity |*m*, *n* > , respectively. **b** Schematic of polarization detection with the proposed MPPC. The MPPC is capable of fully detecting a beam on the HOPS via a single measurement and displaying the SoP in a readable manner with four pointers

Figure 1 schematically shows the detection of a beam on the HOPS using both conventional configurations and our proposed MPPC. It is important to note that traditional configuration involves a series of optical elements such as polarizer, quarter-waveplate (QWP), and mirrors, while our designed MPPC consists solely of a non-interleaved metasurface and a polarizer. As shown in Fig. 1a, two sets of experimental setups are required for detecting the Stokes parameters |2*ψ, 2χ*> and optical singularity |*m*, *n* >, respectively. For detecting the Stokes parameters, we show a time-sharing detection scheme, where a QWP and a polarizer are cascaded and rotated in the detecting process. For detecting the optical singularity, the OAM values of the RCP and LCP components are respectively measured by the triangular aperture technique^55^. Herein, we propose a metrology method for fully detecting beams on the HOPS with miniaturized size, compact detection process, and direct readout mechanism. The designed MPPC, as illustrated in Fig. 1b, demonstrates the capability of complete beam detection on the HOPS via a single measurement and displaying the detected SoP in a readable mechanism.

### Fundamental mechanisms of the MPPC

**Fig. 2:**
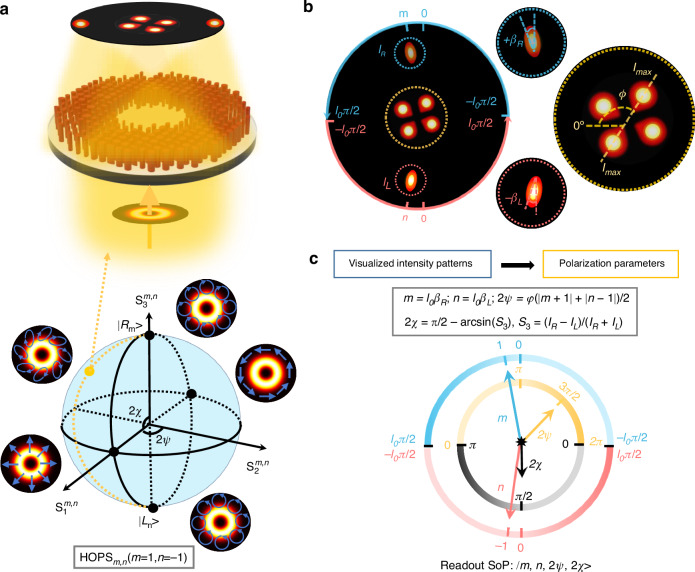
Concept of the metasurface photon polarization clock. **a** Schematic of the designed meta-device capable of converting an incident light beam with unknown SoP into specific output light fields. As an example, a beam (represented as a golden point) located on the surface of a specific HOPS_1,−1_ (1-order) is selected. **b** The top view of the generated light filed in (**a**). An incident beam with different SAM and OAM is transformed into specific focusing patterns, with which one can resolve the full SoP of the incident beam. **c** The schematic of the MPPC, which gives an easy-to-read route for recognizing the incident SoP of the photons, with four pointers showing the four parameters (*m, n, 2ψ, 2χ*) that fully determined an arbitrary SoP

Figure 2a shows the schematic of the designed MPPC capable of fully resolving the SoPs of beams on an arbitrary HOPS_*m,n*_. By converting a light beam with an unknown SoP into specific output light fields, we can determine the four parameters |*m*, *n*, 2*ψ, 2χ*>. Here we choose a beam located on the surface of a specific HOPS_1,−1_ (1-order) as an example. The golden point on the sphere represents the selected beam for detection, with the SoP expresses as |1, −1, *π*/2, π/2>. Figure 2b shows the top view of the generated light filed (|*E*_*x*_|^2^) in Fig. 2(a), in which the generated patterns are divided into two parts, that is, the outer pattern and inner one. The outer pattern consists of two elliptical focusing spots and the inner one shows petal-shaped pattern. To generate this specific light field, the spin-decoupled phase profile is endowed to the MPPC, which is expressed as

4 $${\varphi }_{R}={\rm{arg}}({e}^{0.5{l}_{0}{(\alpha -0.5\pi )}^{2}i}+{e}^{i({\varphi }_{lens}+l\alpha )})$$

5 $${\varphi }_{L}={\rm{arg}}({e}^{0.5{l}_{0}{(\alpha +0.5\pi )}^{2}i}+{e}^{i({\varphi }_{lens}-l\alpha )})$$

where *l*_0_ is the quadric phase coefficient and *α* is the azimuthal angle defined as tan^−1^(*y*/*x*). The first parts on the right-hand side of Eqs. (4) and (5) are combined to construct the phase profile a spin-decoupled angular lens. This lens implements an optical transformation that maps the incident angular momentum (AM) modes into rational focusing patterns on a transverse plane (see section 2 of the Supplementary file). The outer focusing pattern on the transverse plane is divided in half, and the sign of SAM value determines which half the focusing spot located on. As illustrated, both transverse focusing spots show topological charge-dependent azimuthal rotation ranging from *-l*_*0*_*β*_*i*_ to *+l*_*0*_*β*_*i*_, where *β*_*i*_*, i* = *R, L*, represent the orientation angle of these two spots. As a result, the optical singularities represented by the parameters *m* and *n* can be determined by recognizing the azimuthal positions of the two transverse focusing spots. Moreover, the two transverse focusing spots also show ellipticity-dependent intensity (denote as *I*_*R*_ and *I*_*L*_) contrast, from which we can calculate the ellipticity angle *χ*.

The second parts on the right-hand side of Eqs. (4) and (5) are endowed to the meta-device for generating the inner petal-shaped pattern. For this, two spin-decoupled focusing vortex modes are superimposed, where ${\varphi }_{lens}=-2{\rm{\pi }}/{\rm{\lambda }}(\sqrt{{x}^{2}+{y}^{2}+{f}^{2}}-f)$ denotes the phase profile of a lens, where λ is the working wavelength, *l* is the topological charge, and *f* is the focusing distance which equals to the equivalent focal distance of the spin-decoupled angular lens (see Section 2.2 of the Supplementary file). *x* and *y* represent the coordinates in the phase plane. Here, we set *l* = 1, thus, under linear polarized light illumination, two petal-shaped intensity patterns are generated due to superposition of two focused CP vortex beams possessing identical topological charges but opposite signs^56^. Hence, for a selected SoP on the 1-order HOPS, four petals appear in its final inner pattern based on AM’s superposition rule. As shown in the right panel of Fig. 2(b), the resulting petal-shaped pattern exhibits polarization azimuth angle-dependent self-axis rotation. By analyzing its orientation angle, the polarization azimuth angle *ψ* can be determined. Therefore, by analyzing characteristics of generated intensity distributions, an incident beam’s SoP can be fully determined as |*m*, *n*, 2*ψ*, 2*χ*>. The schematic of the MPPC is illustrated in Fig. 2c, where SoP can be directly readout using four pointers, which analogous to a conventional clock displaying precise time via three pointers.

### Implementation of the MPPC

Owing to the unprecedent capacity of wavefront manipulation, a metasurface can integrate the functions of spin-decoupled angular lens and focusing vortexes into a single monolithic device for fully polarization detection. Then, the key issue is the design of a spin-decoupled metasurface capable of independently controlling the LCP and RCP spin eigenstates (with the Jones vectors denotes as |*L* > = [1, i]^*T*^ and |*R* > = [1, −i]^*T*^). In other words, the spin-decoupled metasurface (denoted by the Jones matrix *J*(*x,y*)) is able to impart two independent phase profiles *φ*_*L*_(*x,y*) and *φ*_*R*_(*x,y*) on the two output spin eigenstates, respectively. Therefore, the interaction between light and metasurface can be expressed as $J(x,y)|R\rangle =\exp [i{\varphi }_{L}(x,y)]|L\rangle$ and $J(x,y)|L\rangle =\exp [i{\varphi }_{R}(x,y)]|R\rangle$, where *x* and *y* represent the coordinates of meta-atoms in the metasurface plane. To obtain the spin-decoupled metasurface, the propagation phase and geometric phase are combined to enable the conversion of a spin eigenstate to its spin flipped counterpart while achieving complete 2π phase coverage^5,34^. The propagation phase commonly represents the phase shifts *δ*_*x*_ and *δy* along the two perpendicular symmetry axes of the meta-atom. The geometric phase is governed by the in-plane orientation angle *θ* of the meta-atom. Hence, a metasurface with spin-decoupled phase control can be expressed as

6 $${\varphi }_{R}(x,y)={\delta }_{x}(x,y)-2\theta (x,y)$$

7 $${\varphi }_{L}(x,y)={\delta }_{y}(x,y)+2\theta (x,y)-\pi$$

8 $$\theta (x,y)=[{\varphi }_{R}(x,y)-{\varphi }_{L}(x,y)]/4$$

**Fig. 3:**
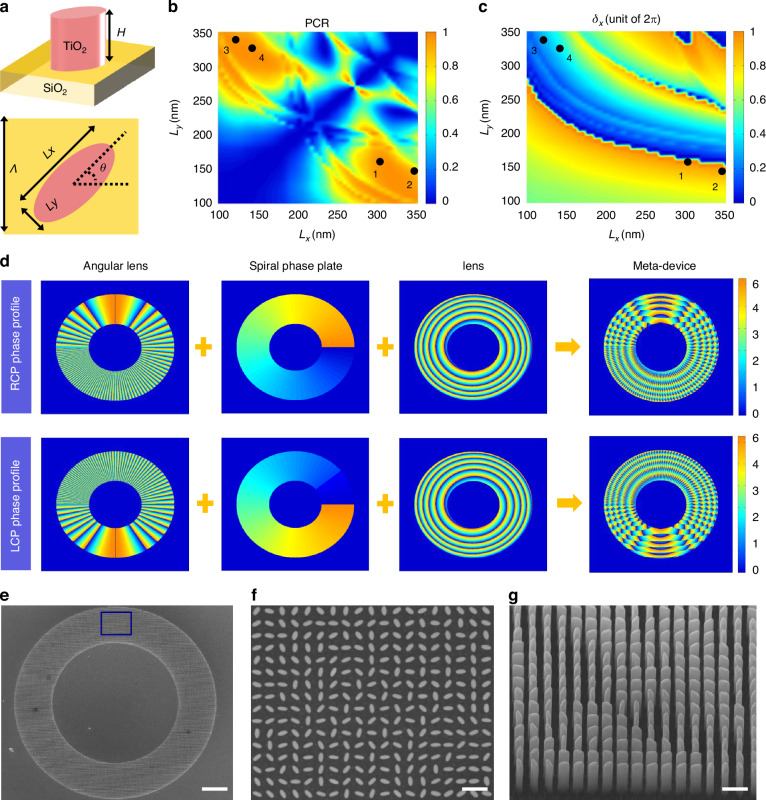
Design and SEM images of the MPPC. **a** Side view and top view of the meta-atom, which composed of an elliptical TiO_2_ nanoblock on the SiO_2_ substrate. **b** Calculated PCR as a function of nanoblock’s in-plane dimensions at the working wavelength of 633 nm. **c** Simulated phase shifts as a function of nanoblock’s in-plane dimensions under *x*-linearly polarized incident light. The black dots in (**b**) and (**c**) denote the selected four fundamental nanoblocks, which simultaneously owns a phase gradient of π/4 and a relatively high PCR. **d** The phase profile components of the MPPC. The phase profiles of the RCP and LCP components are a sum of an angular lens, a spiral phase plate and a lens, respectively. **e** Overall SEM image of the meta-device. Scale bar: 30μm. **f**, **g** Top view and side view SEM images of part of the sample that marked with the green frame in (**e**). Scale bar: 1μm

Therefore, the implementation of *J*(*x*, *y*) can be achieved through meticulous design of meta-atoms, which can provide the required three values (*δ*_x_, *δ*_y_ and *θ*) at any coordinate (*x, y*). Figure 3a illustrates the side and top views of the designed meta-atom comprising a TiO_2_ elliptical nanoblock placed on a silica substrate. The detailed parameters include a subwavelength lattice constant *Λ* = 450 nm, height *H* = 1000 nm and in-plane dimensions (major axis *L*_*x*_, minor axis *L*_*y*_ and the orientation angle *θ*). Full-wave finite-difference time-domain simulations were conducted to investigate the transmission properties of the meta-atoms (see details in section 3 of the Supplementary file). Based on these simulation results, Fig. 3b presents the calculated polarization conversion rate (PCR) as a function of the meta-atoms’ diameters (*L*_*x*_ and *L*_*y*_) at the working wavelength of *λ* = 633 nm. Additionally, Fig. 3c showcases the corresponding simulated phase shifts under *x*-linearly polarized incident light. Four fundamental meta-atoms with relatively high PCR values and a phase difference of π/4 were selected as denoted by black dots in Fig. 3b, c. These four basic meta-toms along with their mirror counterparts are combined to provide eight phase levels encompassing the whole 2π phase range. Figure 3d shows the spin-decoupled phase profiles components of the MPPC. The phase profiles of the RCP and LCP components are a sum of an angular lens, a spiral phase plate and a lens, respectively. Subsequently, these imparted phase profiles are integrated into spin-decoupled metasurface to form the final meta-device. Through a phase-matching process, the in-plane dimensions and orientation angle of the meta-atom at each point of the final metasurface MPPC can be determined.

Then, the metasurface sample is fabricated using e-beam lithography and atomic layer deposition, following the now-standard procedure (see Materials and Methods for fabrication details). The detailed parameters of the ring-shaped MPPC sample are as follows. Here, *l*_0_ is set as 40 to obtain a large OAM mode recognition space of *l*_0_*π*. The inner and outer radii of the ring-shaped PPC are set as *r*_i_ = 75 μm and *r*_o_ = 120 μm (see section 4 of the Supplementary file). The corresponding focal distance of the MPPC is calculated as *f* = 2.36 mm. Scanning electron microscopy (SEM) images of the fabricated sample are shown in Fig. 3e–g. Figure 3e shows an overall SEM image of the meta-device, while Fig. 3f, g present top view and side view SEM images specifically focusing on locally amplified regions within the sample.

### Fully polarization resolving on arbitrary HOPS by the designed MPPC

**Fig. 4:**
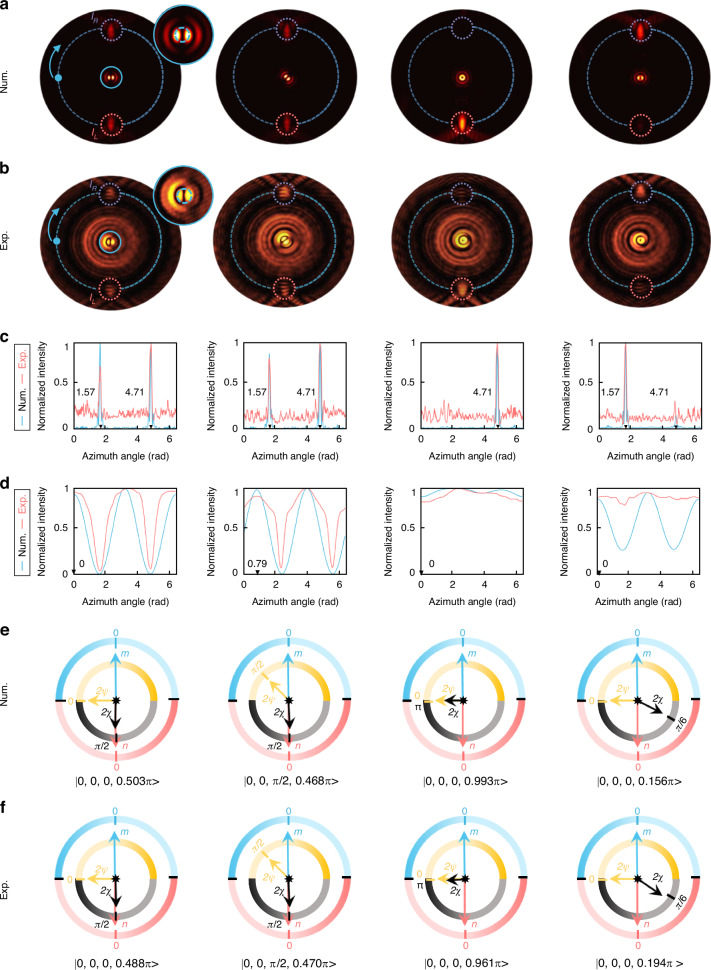
Resolving SoP of beams on the HOPS_0,0_ by using the MPPC. **a**, **b** Simulated and measured intensity (|*E*_*x*_|^2^) profiles of the MPPC for incident light with four SoPs from left to right: |0, 0, 0, π/2>, |0, 0, π/2, π/2>, |0, 0, 0, π>, and |0, 0, 0, π/6>. **c**. The normalized intensity distributions along the turquoise dashed ring shown in (**a**) and (**b**). The turquoise and rose red solid lines represent the simulated and measured results, respectively. **d** The normalized intensity distributions along the turquoise dashed ring shown in the enlarged insets of (**a**) and (**b**). The turquoise and rose red solid lines represent the simulated and measured results, respectively. The black triangle marks in (**c**) and (**d**) represent the azimuth angle of the intensity peaks. **e**, **f** Numerically and experimentally retrieved SoPs by the MPPC, where the SoPs can be directly readout by four pointers

To validate the functionality of the proposed MPPC, various beams on different order HOPS_*m*,*n*_ are taken into consideration. Initially, we focus on fully resolving the SoPs on the 0-order HOPS, i.e. the standard PS denoted as HOPS_0,0_. Figure 4a, b presents the numerical and measured intensity (|*E*_*x*_|^2^) profiles of the MPPC under different incident beams on the HOPS_0,0_, respectively. The incident beams with eight SoPs located on the surface of the HOPS_0,0_ are chose as typical examples. Here we demonstrated fully resolving four beams, with the certain SoPs are |0, 0, 0, π/2>, |0, 0, π/2, π/2>, |0, 0, 0, π>, and |0, 0, 0, π/6> (results for resolving other four beams can be found in Figure S6 of Supplementary file). To characterize the functionality of the designed MPPC, we adopt a specific experimental set-up (see section 5 of the Supplementary file). Figure 4c depicts intensity distributions along the green and red dashed rings (starting from leftmost point and moving clockwise) in Fig. 4a, b, respectively. The presence of focusing spots at top and bottom parts indicates SAM values +1 and −1, respectively. The orientation angle determines topological charge of the two CP components, which expressed as *l*_*i*_ = *l*_*0*_*β*_*i*_, *i* = *R*, *L*. It is observed that, for all the cases, the two transverse focusing spots exhibit at azimuthal angle of *β*_*R*_ = *β*_*L*_ = 0 which implies *m* = *n* = 0. The two transverse focusing spots also show ellipticity-dependent intensity contrast (denoted as *I*_*R*_ and *I*_*L*_), enabling the calculation of the ellipticity angle 2*χ* (see section 6.1 of the Supplementary file).

The intensity patterns in the center focal planes result from the superposition of the total generated AM modes. An arbitrary polarized light beam can be decomposed into a superposition of two CP modes, represented as ${e}^{i{\varphi }_{0}}|R\rangle +{e}^{-i{\varphi }_{0}}|L\rangle$, where *φ*_0_ is the relative phase difference between the two CP states. The final superimposed mode can be characterized by passing through a linear polarizer that forms an angle *φ* with respect to the *x*-axis. The transmitted intensity profile can be expressed as $E=[1+co{s}^{2}(la/2+{\varphi }_{0}+\varphi )]$, where *l* = *|m* + 1 | *+|n*−1*|* is the total topological charges of the OAM modes. The maximum transmitted intensity values occur at the azimuth angle ${\varphi }_{max}=(2N\pi -2{\varphi }_{0}-2\varphi )/|l|$, where $N=0,1,2\ldots |l|-1$. Figure 4d depicts the intensity distributions along the green and red dashed rings in the enlarged insets of Fig. 4a, 4b, respectively. It can be observed that both the simulated and measured intensity profiles exhibit maximum at specific azimuth angle, allowing us to identify these angles as $2\psi =\varphi (|m+1|+|n-1|)/2$. Noting that, for the two CP modes, the intensity distributions exhibit doughnut-shaped, indicating a polarization azimuth angle *ψ* = 0. Therefore, by using the MPPC, all four parameters |*m*, *n*, *2ψ*, 2*χ*> of an arbitrary beam on the HOPS_0,0_ can be fully determined via a single measurement. Figure 4e, f shows the numerically and experimentally reconstructed SoPs using MPPC, in which SoPs can be directly readout via four independent pointers. The numerically and experimentally reconstructed SoPs show well agreement with the original ones (see section 6.2 of the Supplementary file). The experimental average SoP reconstruction error is 2.3%, which shows comparable performance to the current polarization detection meta-device. Due to the specific ring-shaped structure of the MPPC, part of the incident beam directly passing through the central of the meta-device, and comes to be the unwanted energy distributions. Moreover, the ring shape meta-device structure will lead to unavoidable diffraction. The presence of these two undesired background noises, in addition to deviations caused by fabrication and errors in measurement, ultimately results in small disparities between the numerical and experimental results. To solve this issue, we can fill the center area of the MPPC with meta-atoms that exhibit nearly zero transmission under arbitrary incident polarized light.

**Fig. 5:**
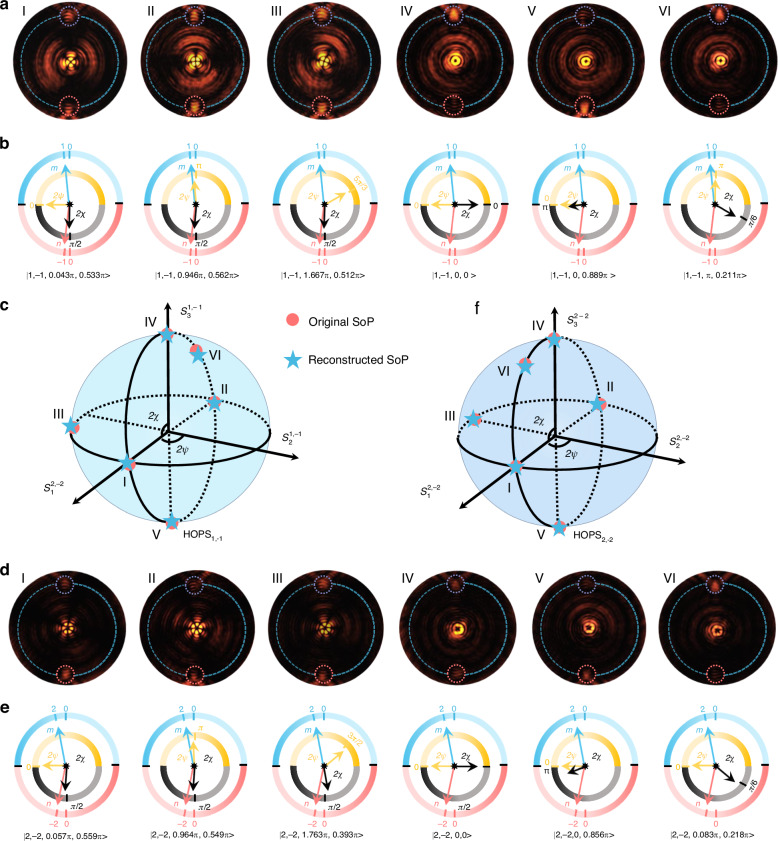
Resolving SoP of beams on the HOPS_1,−1_ and HOPS_2,−2_. **a** Measured intensity (|*E*_*x*_|^2^) profiles of the MPPC for incident light with six SoPs from left to right: |1, −1, 0, π/2>, |1, −1, π, π/2>, |1, −1, 5π/3, π/2>, |1, −1, 0, 0>, |1, −1, 0, π>, and |1, −1, π, π/6>. **b** Experimentally retrieved SoPs by the MPPC, where the SoPs can be directly readout by four pointers. **c** Original (red points) and reconstructed (green asterisks) SoPs represented on the HOPS_1,−1_. **d** Measured intensity (|*E*_*x*_|^2^) profiles of the PPC for incident light with six SoPs from left to right: |2, −2, 0, π/2>, |2, −2, π, π/2>, |2, −2, 3π/2, π/2>, |2, −2, 0, 0>, |2, −2, 0, π>, and |2, −2, 0, π/6>. **e** Experimentally retrieved SoPs by the MPPC, where the SoPs can be directly readout by four pointers. **f** Original (red points) and reconstructed (green asterisks) SoPs represented on the HOPS_2,−2_

Subsequently, we proceed to fully resolve the SoPs on HOPS_1,−1_ and HOPS_2,−2_ by using the MPPC, as depicted in Fig. 5. Without loss of generality, we picked up six different vector states on the HOPSs to prove our designed MPPC. Here, we present only the experimental results, while detailed numerical results can be found in section 7 of the Supplementary file. Figure 5a shows the measured intensity profiles of the MPPC under different incident beams on the HOPS_1,−1_. The six SoPs are |1, −1, 0, π/2 > , |1, −1, π, π/2>, |1, −1, 5π/3, π/2>, |1, −1, 0, 0>, |1, −1, 0, π>, and |1, −1, π, π/6>. By analyzing the generated intensity patterns, we can retrieve the four parameters |*m*, *n*, *2ψ*, 2*χ* > (see details in Fig. S11 of the Supplementary file). As shown in Fig. 5b, the reconstructed six SoPs are presented in a readable mechanism by the MPPC with four independent pointers. To visually represent the outcomes, Fig. 5c shows the representation of both original and reconstructed SoPs on the HOPS_1,−1_. Additionally, Fig. 5d shows the measured intensity profiles of the MPPC under different incident beams on the HOPS_2,−2_. The six SoPs are |2, −2, 0, π/2>, |2, −2, π, π/2>, |2, −2, 3π/2, π/2>, |2, −2, 0, 0>, |2, −2, 0, π>, and |2, −2, 0, π/6>. Similarly, the four parameters |*m*, *n*, *2ψ*, 2*χ*> of an arbitrary beam on the HOPS_2,−2_ can be retrieved by analyzing the generated intensity patterns (see details in Figure S12 of the Supplementary file). The six SoPs can also be readout by the MPPC with four independent pointers, as illustrated in Fig. 5e. Figure 5f shows the representation of the original and reconstructed SoPs on the HOPS_2,−2_. For beams on both HOPSs, the experimentally reconstructed SoPs show good agreement with the original ones (see details in section 7 of the Supplementary file). The experimental average SoP reconstruction errors for beams on the 1st and 2nd HOPSs are 4.6% and 11.1%, which show comparable performance to the current polarization detection meta-device.

## Discussion

To further validate the functionality of the designed MPPC, we also numerically demonstrate fully resolving the SoPs on the hybrid-order HOPS_2,−1_ and 5th order HOPS (see section 8 of the Supplementary file), respectively. The numerically reconstructed SoPs show excellent agreement with the original ones. Here, by using the designed MPPC, we demonstrate fully resolving the SoPs on the HOPS_0,0_, HOPS_1,−1_, HOPS_2,−2,_ and the hybrid-order HOPS_2,−1_, respectively. The proposed polarization metrology method is general and can be extended for fully detecting beams on an arbitrary HOPS.

Currently, vector beams (VBs) have raised a range of important applications in various fields such as optical communication, display technology and encryption methods, particle manipulation techniques, laser direct writing, high-dimensional quantum information processing. Here we rehearse a VB optical communication system that enables the utilization of VBs positioned at arbitrary locations on the HOPS, relying on the pioneering work that focus on VB communication^57^. In this system, by using our designed MPPC as calibration device and decoding device, the vector beams can be treated as independent information carriers for free-space optical communication (see details in section 10 of the Supplementary file). Based on the spatial polarization differential phase shift keying protocol that encode information in the relative phase between the two polarization components of a vector beam, our proposed VB optical communication system can be used for high-capacity information processing.

In summary, we have overcome the restrictions of contemporary polarization detecting strategy and proposed a metrology method capable of fully detecting SoPs on any given HOPS. This novel metrology method is implemented by a single layer non-interleaved metasurface, which transforms optical singularities and Stokes parameters into specific intensity distributions on a transverse plane. Via a single measurement, the designed MPPC is able to fully detecting SoPs on an arbitrary HOPS with a direct readout mechanism. As proof of concept, we theoretically and experimentally demonstrate the fully detection of SoPs on HOPS_0,0_, HOPS_1,−1_ and HOPS_2,−2_. Moreover, fully resolving the SoPs on the hybrid-order HOPS_2,−1_ and HOPS_5,-5_ are theoretically demonstrate as well. Noting that, although our MPPC operating in the visible frequency, this design strategy can be readily applied to other frequencies. The MPPC provides a fire-new approach for detecting the SoPs with miniaturized size, simple detection process, and direct readout mechanism, holding significant potential applications in various fields such as remote sensing, optical trapping, material analysis, and high-dimensional quantum technology.

## Materials and methods

### Numerical simulations

Numerical simulations of the meta-atoms were carried out using the finite difference time domain method. The period of meta-atoms was set as 450 nm. The substrate was included in the simulations. The refractive index of SiO_2_ was taken as 1.46 at the operating wavelength of 633 nm. The refractive index of the TiO_2_ was the measurement result by the ellipsometer.

### Device fabrication

The samples were fabricated using electron beam lithography (EBL) along with the etching technique. First, a 1000-nm-thick polymethyl methacrylate (PMMA) electron-beam resist layer was spin-coated at 2000 rpm on the transparent silica substrate with an ITO film layer and baked on a hot plate for 4 min at 180 °C. Next, the sample was exposed by EBL with a 100-KV voltage and a beam current of 200 pA. Subsequently, we put the exposed sample in a mixed solution of isopropanol and methyl isobutyl ketone (IPA: MIBK = 3:1) for 1 minute, and then fixed it in the IPA solution for 1 minute at room temperature. Later, we used the atomic layer deposition (ALD) system to fill the exposed area with 200 nm TiO_2_. We use PMMA for the positive photoresist exposure process, which is void before deposition. Then the deposited thickness of TiO_2_ is related to the semi-minor axis of the maximum meta-atom. After this process, there will be a layer of 200 nm TiO_2_ on the top of the entire sample, and we removed it by ion beam etching (IBE) in the next process. After removing the TiO_2_ on the top layer, we used reactive ion etching (RIE) to remove the resist. Finally, the TiO_2_ nanostructures with a high aspect ratio (of up to 10) are obtained.

## Supplementary information

Supplementary Information for Metasurface Higher-Order Poincaré Sphere Polarization Detection Clock

## Data Availability

Data underlying the results presented in this paper are available from the corresponding author upon reasonable request.
