# Supplementary material for: Metasurface higher-order poincaré sphere polarization detection clock
Source: Light Sci Appl. 2025 Jan 26;14:63. doi: 10.1038/s41377-024-01738-1 (PMC11762790; doi:10.1038/s41377-024-01738-1)
Supplement: Supplementary file 1 — Supplementary Information for Metasurface Higher-Order Poincaré Sphere Polarization Detection Clock [file 41377_2024_1738_MOESM1_ESM.docx]

**Supplementary Material for**

**Metasurface Higher-Order Poincaré Sphere Polarization Detection Clock**

Hui Yang^1,2,#^, Kai Ou^3,#^_,_ Qiang Liu^1^, Meiyu Peng^2^, Zhenwei Xie^4^, Yuting Jiang^1^, Honghui Jia^1,5^, Xinbin Cheng^3^, Hui Jing^2^, Yueqiang Hu^1,5,*^, and Huigao Duan^1,5,*^

^1^National Research Center for High-Efficiency Grinding, College of Mechanical and Vehicle Engineering, Hunan University, Changsha 410082, China

^2^School of Physics and Electronics, Hunan Normal University, Changsha, 410081, China

^3^Institute of Precision Optical Engineering, School of Physics Science and Engineering, Tongji University, Shanghai 200092, China

^4^Nanophotonics Research Center, Shenzhen Key Laboratory of Micro-scale Optical Information Technology, Institute of Microscale Optoelectronics, Shenzhen University, Shenzhen 518060, Guangdong, China

^5^Greater Bay Area Institute for Innovation, Hunan University, Guangzhou 511300, Guangdong Province, China

^#^The authors contribute equally to this work.

**Corresponding authors*: huyq@hnu.edu.cn; duanhg@hnu.edu.cn

1. **Description of the higher-order Stokes parameters**

For representing lights with spatially inhomogeneous polarizations such as vector vortex beams, the extended generalized geometric representation known as a higher-order Poincaré sphere (HOPS) is adopted [1, 2]. For an arbitrary beam located on the surface of the HOPS, the higher-order Stokes parameters in the sphere’s Cartesian coordinates can be derived as

$S_{0}^{m,n}{=\left| E_{R}^{m} \right|}^{2}+\left| E_{L}^{n} \right|^{2}$ (S1)

$S_{1}^{m,n}=2\left| E_{R}^{m} \right|\left| E_{L}^{mn} \right|cos\varphi$ (S2)

$S_{2}^{m,n}=2\left| E_{R}^{m} \right|\left| E_{L}^{mn} \right|sin\varphi$ (S3)

$S_{3}^{m,n}{=\left| E_{R}^{m} \right|}^{2}-\left| E_{L}^{n} \right|^{2}$ (S4)

where $E_{R}^{m}$ and $E_{L}^{n}$ are the complex amplitudes of the two circularly polarized (CP )vortex bases, which are expressed as |$R_{m}$>$=e^{im\varphi}$|*R*> and |$L_{n}$>$=e^{in\varphi}$|*L*>. The integer *m* and *n* denote the topological charges of the two CP vortex beams. $\varphi=\arg\left( E_{R}^{m} \right)-arg(E_{L}^{n})$ is the phase difference between the two CP vortex beams. Noting that, when *m* = *n* = 0, the HOPS degrades into the standard PS, and the higher-order Stokes parameters changes to the standard ones as well. Hence, the standard PS can be treated as a particular case of the HOPS, that is, the 0-order HOPS. Using the Eqs. S1-S4, an arbitrary beam with space-variant SoP can be mapped to the surface of the HOPS with spherical coordinates (2*ψ,* 2*χ*).

1. **Principle of the spin-decoupled angular lens**

**2.1 Design of the angular lens**

The angular lens performs an optical transformation that maps the incident orbital angular momentum (OAM) modes into rational focusing patterns on a transverse plane [3, 4]. The angular lens owns an azimuthal-quadratic phase profile, which can be expressed as

$\varphi_{(\alpha)}=0.5l_{0}\alpha^{2}$ (S5)

where *l*_0_ is the quadric phase coefficient and *α* is the azimuthal angle defined as tan^-1^(*y*/*x*). For an incident OAM beam with a topological charge of *l* illuminates on the angular lens, the accumulated phase profile can be derived as

$\varphi_{(\alpha)}+l\alpha=0.5l_{0}{(\alpha+l/l_{0})}^{2}-l^{2}/(2l_{0})=\varphi_{(\alpha^{'})}-l^{2}/(2l_{0})$ (S6)

where $\alpha^{'}$ denote a new azimuthal angle. Neglecting the azimuth-independent term on the right side of Eq. S6, we can get

$\varphi_{(\alpha^{'})}=0.5l_{0}{(\alpha+l/l_{0})}^{2}$ (S7)

Comparing Eq. S5 with Eq. S7, we find that there is only a mode-dependent azimuthal rotation through the angular lens:

$\alpha_{l}=-l/l_{0}$ (S8)

A phenomenological explanation of the azimuthal rotation is illustrated in Fig. S1, illustrating the accumulated phase profiles and focusing behaviors after different OAM modes transmitting through the angular lens are depicted. A typical azimuthal-quadratic phase profile is shown in Fig. S1a. It can be observed that phase profile is symmetric along the *y*-axis and changes rapidly with the azimuthal angles especially approaching ±π. Fig. S1d shows the corresponding calculated intensity on the focal plane, from which a focusing pattern exhibits at the azimuthal angle of $\alpha_{l}$= 0 under a normally incident plane wave (with OAM *l* = 0). The total phase profile and focusing pattern both exhibit a clockwise rotation when a normally incident light beam carrying an OAM mode (*l* = 20) interacts with the angular lens, as depicted in Fig. S1b and S1e. Moreover, when we change the OAM mode to *l* = -20, the total phase profile and focusing pattern both rotate anti-clockwise, as shown in Fig. S1c and S1f. As a result, the OAM modes with opposite sign are focused symmetric about azimuthal angle $\alpha$ = 0, which agrees well with Eq. S8. Moreover, in the whole process, the focusing pattern rotates clockwise with an azimuthal interval of 1/*l*_0_ as the topological charge increases from negative to positive values. Hence, by recording the azimuthal angle of the resulting focusing pattern, we can accurately determine the incident OAM mode as

$l=-l_{0}\alpha_{l},\alpha_{l}\epsilon[-\pi,\pi]$ (S9)


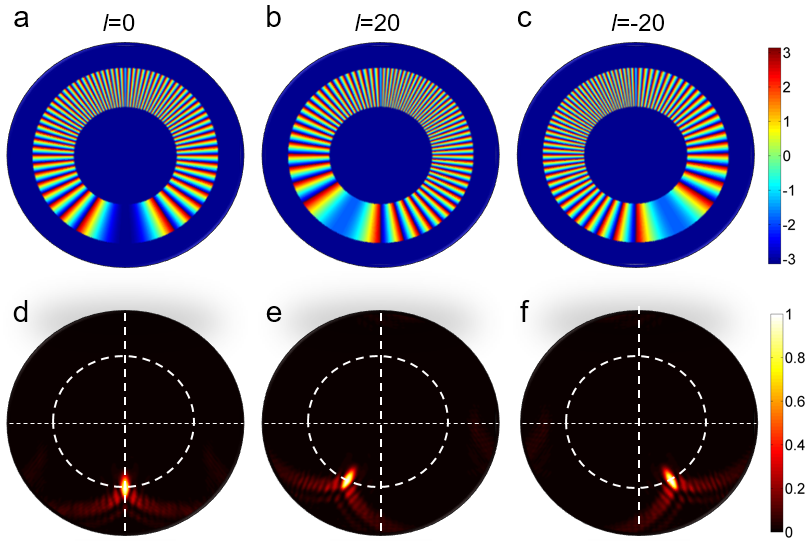


Fig. S1. The phenomenological explanation of the angular lens. (a-c) Accumulated phase profiles after different OAM modes transmitting through the angular: (a) *l* = 0, (b) *l* = 20, and (c) *l* = -20. (d-f) The corresponding calculated focusing intensities in the cross plane. The quadric phase coefficient is set as *l*_0_=40.

**2.2 Deduction focal distance of the angular lens**

From Eq. S5, we cannot directly deduce the focal distance of the angular lens. As illustrated in Fig. S2, by flattening the Polar coordinates into Cartesian coordinates, we can translate an azimuthal-quadratic phase profile (Eq. S5) into a cylindrical-quadratic phase profile as

$\varphi_{(r)}=0.5l_{0}{(r/r_{0})}^{2}$ (S10)

Therefore, comparing the flattened phase profile with the phase profile of a cylindrical Fourier lens, the focal distance can be easily determined and expressed as

$\varphi_{(r)}=0.5l_{0}{(r/r_{0})}^{2}=k_{0}r^{2}/(2f)$ (S11)

where *k*_0_=*2π*/*λ* is the wavevector in free space, *f* is the focal distance and *r*_0_ is the radius of the angular lens. From Eq. S11, the focal distance can be deduced as

$f=k_{0}r_{0}^{2}/l_{0}$ (S12)

where $r_{0}=(r_{i}+r_{o})/2$and $r_{i}$ and $r_{o}$ represent the inner and outer radii of the angular lens.


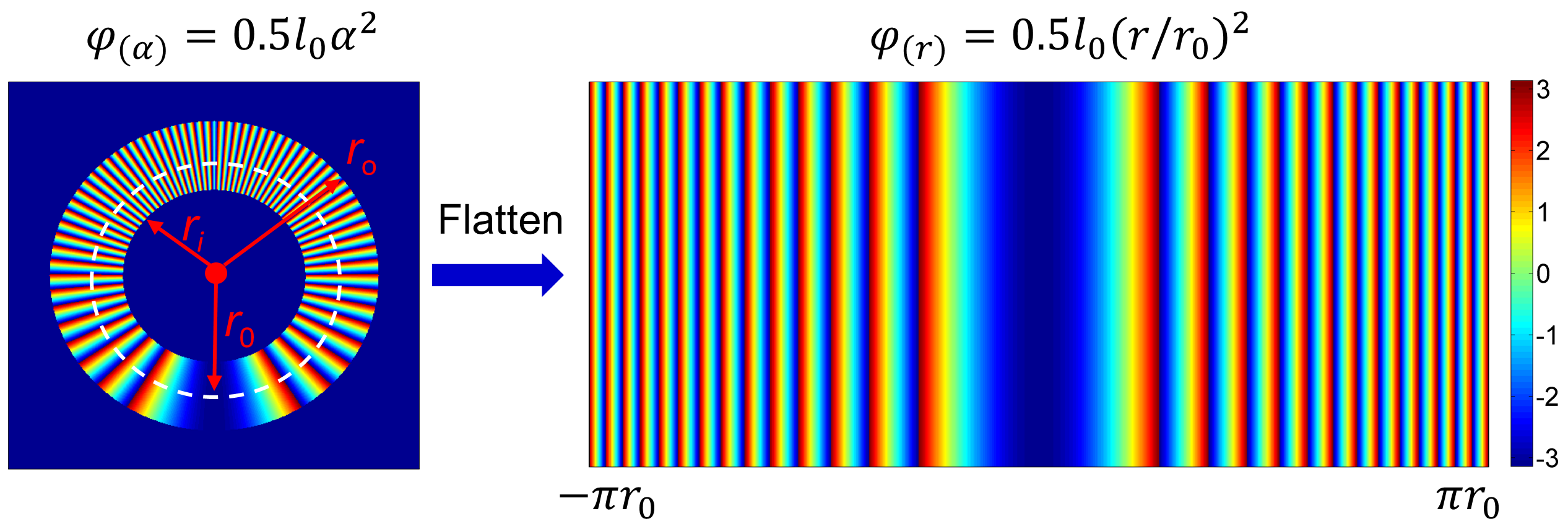


Fig. S2 Flatten the phase profile of the angular lens in Polar coordinates into Cartesian coordinates. The azimuthal-quadratic phase profile (Left panel) is flatten to cylindrical-quadratic phase profile (Right panel). The other parameters are the quadric phase coefficient *l*_0_ = 40, the radii of the angular lens $r_{i}=75 \mu m \mathrm{and} r_{o}=120 \mu m$.

**2.3 Design of the spin-decoupled angular lens**

The above-discussed angular lens can only detect the OAM modes of incident beam. As we know, light beam is able to carrying both the spin angular momentum (SAM) and OAM modes. In order to detect both SAM and OAM modes simultaneously, a spin-decoupled angular lens is required. For this, the incident left-handed circularly polarized (LCP) and right-handed circularly polarized (RCP) beams respectively picks up azimuthal-quadratic phase profiles as

$\varphi_{R}=0.5l_{0}{(\alpha-0.5\pi)}^{2}$ (S13)

$\varphi_{L}=0.5l_{0}{(\alpha+0.5\pi)}^{2}$ (S14)

From Eq. S13 and Eq. S14, the calculated intensity profiles on the focal plane are shown in Fig. S3. Here, we choose the linearly polarized vortex beams as typical examples, which can be treated as the superimposition of LCP and RCP vortex beams with identical topological charge. The topological charge is ranging from *l* =-15 to *l* = 20 with a step length of *l* = 5. It can be observed that there are two transverse focal spots on the focal plane, positioned on either side of the x-axis (excluding the case where both are located on the *x*-axis when *l* = 0). Therefore, SAM values can be easily determined by inspecting which half of the circle the focal spot is located on. Moreover, the two focusing spots show topological charge-dependent azimuthal rotation. As a result, the optical singularities represented by the parameters *m* and *n* can be determined by recognizing the azimuthal positions of the two transverse focusing spots.


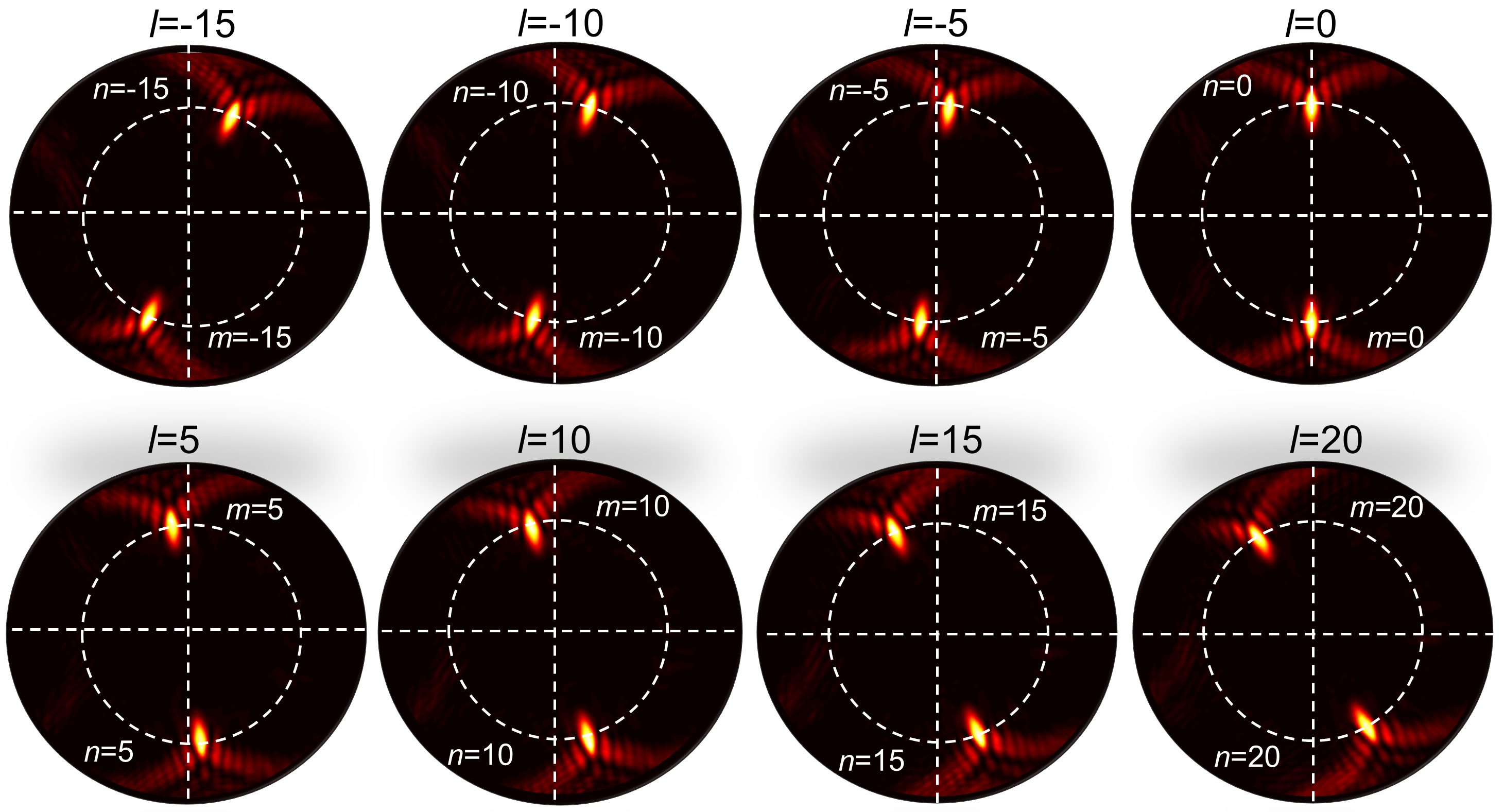


Fig. S3. The calculated intensity profiles on the focal plane of a spin-decoupled angular lens with linearly polarized vortex beams carrying different topological charges. The topological charge is ranging from *l* = -15 to *l* = 20 with a step length of *l* = 5. The other parameters are the quadric phase coefficient *l*_0_ = 40, the radii of the spin-decoupled angular lens are $r_{i}=75\mu m,r_{o}=120\mu m$.

**3. The design of the meta-atoms**


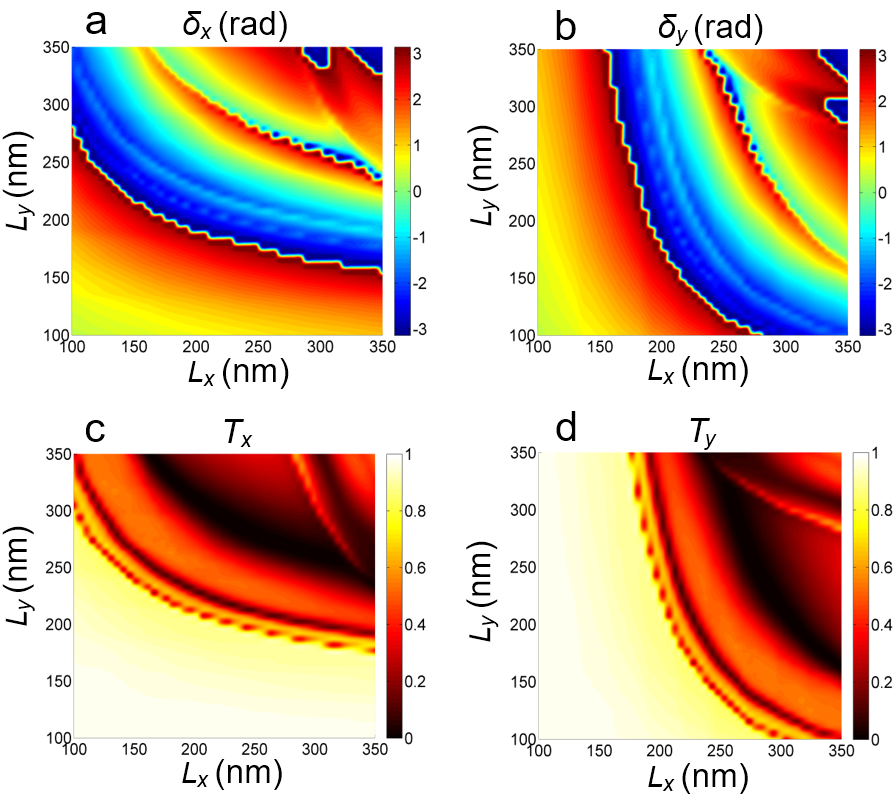


Fig. S4 (a) and (c) Phase shift and transmission as a function of meta-atom’s major and minor axes with *x*-linearly polarized (XLP) incident light. (b) and (d) Phase shift and transmission as a function of meta-atom’s major and minor axes with *y*-linearly polarized (YLP) incident light. In consideration of the practical manufacturing process, elliptical nanoblocks with lengths (*L_x_* and *L_y_*) range from 100 nm to 350 nm are used.

In order to investigate the transmission properties of the meta-atom, full-wave simulations were performed using the finite-difference time-domain (FDTD) method. Figures S4(a-d) demonstrate the transmission properties of the meta-atoms, in which the simulated phase shifts and transmission of the meta-atoms as functions the major and minor axes of the meta-atom for the *x*- and *y*-linearly polarization (LP) incident lights. In these simulations, periodic boundary conditions are used along the *x*- and *y*-axes, and the perfectly-matched layer conditions are used along the *z* axis. The results show that by varying the lateral dimension of the meta-atom from minimum to maximum, a required phase coverage of 0-2π can be achieved. Then, by using Eqs. 6-8 in the main text, one can arbitrarily and independently manipulate the phase shifts of the two spin eigenstates by proper designing the three parameters of the meta-atom (*L_x_*, *L_y_* and *θ*).

**4. Radius setting of the spin-decoupled angular lens**

To obtain well transverse focusing patterns through the spin-decoupled angular lens, it is crucial to optimize the radius of this spin-decoupled angular lens. Here, we have chosen a linearly polarized vortex beam with a topological charge of *l* = 5 as an illustrative example. The calculated focusing patterns of the spin-decoupled angular lens with the same coefficient *l*_0_ = 40 but different radius are illustrated in Fig. S5. When the inner radius is set to 0 μm (the spin-decoupled angular lens changes to a common circular shape), the intensity profiles of LCP and RCP spread along the azimuth and form two sweat-heart shaped patterns. In this case, most of the intensity is dispersed and not well focused. It can be observed that the intensity spreading is gradually suppressed as the increment of the inner radius. Then, as the inner radius reaches 75 μm, the intensity profiles of LCP and RCP show well transverse focal spots. With a further increment of the inner radius, the intensity spreading phenomenon emerges again. Therefore, to obtain well transverse focusing patterns, the inner radius is selected as 75 μm when the outer radius is 120 μm. Note that, when the inner radius and outer radius are simultaneously scaled with a factor, the focusing patterns still keep good (show the case of $r_{i}=50\mu m,r_{o}=80\mu m$).

Moreover, the variation in both the inner and outer radii of the annular metasurface has an impact on the size of the central spot. The generation of the inner petal-shaped pattern involves the superposition of two spin-decoupled focusing vortex modes, as discussed in the main text, to design the MPPC. As a result, the size of the central petal-shaped pattern depends on the numerical aperture of the focusing vortex metalens. Even so, the shape of the central pattern stays the same. Hence, it does not affect the determination of the polarization azimuth angle *ψ* and we focus on optimizing both the inner and outer radius to achieve a nice transverse focusing pattern.


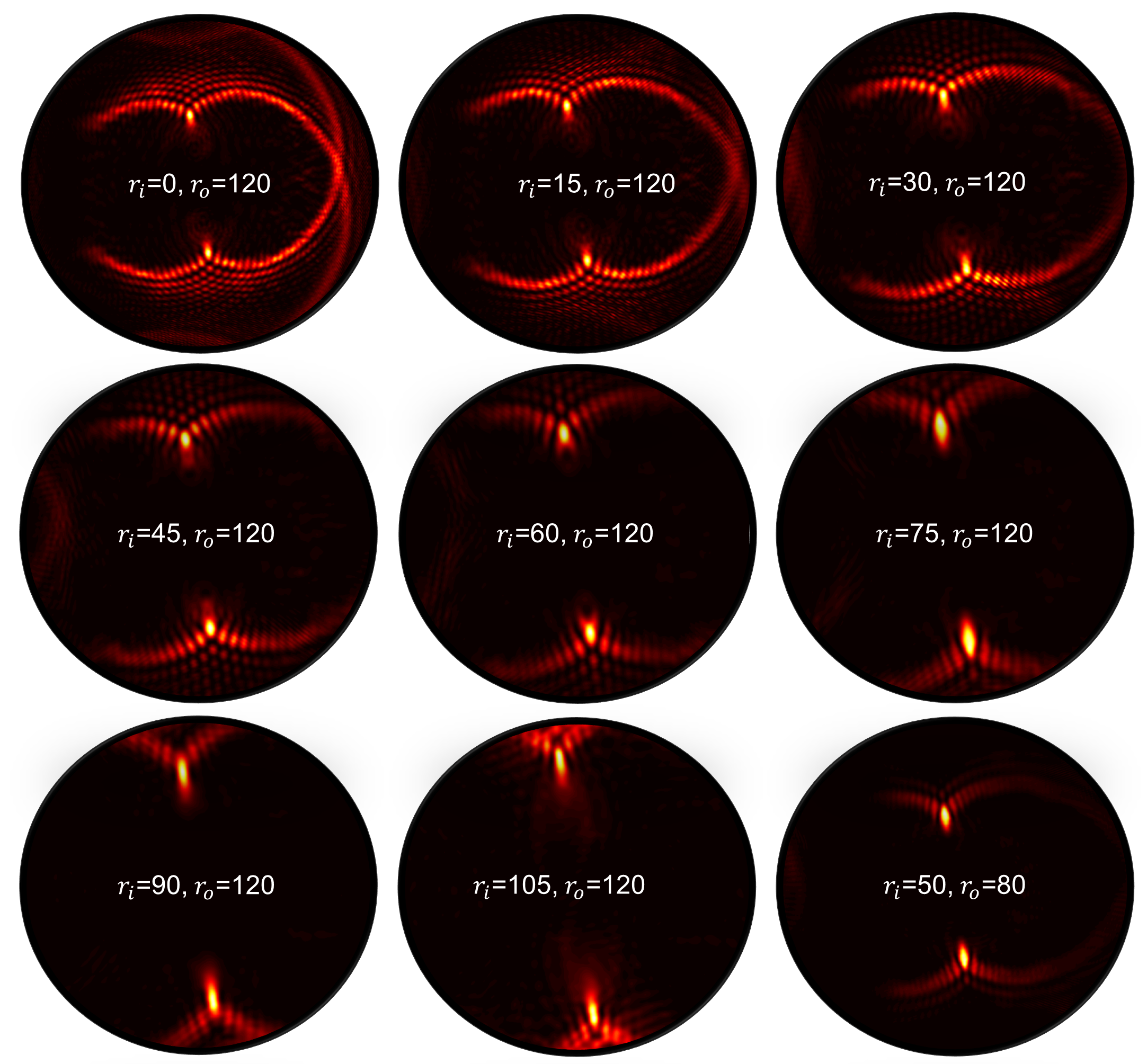


Fig. S5. The calculated intensity profiles on the focal plane of a spin-decoupled angular lens with different radii. The length unit of the radii is micrometer. The linearly polarized vortex beams carrying topological charge of *l* = 5, namely, *m* = *n* = 5, is chose in the simulation.

**5. Experimental set-up for SoPs characterization**


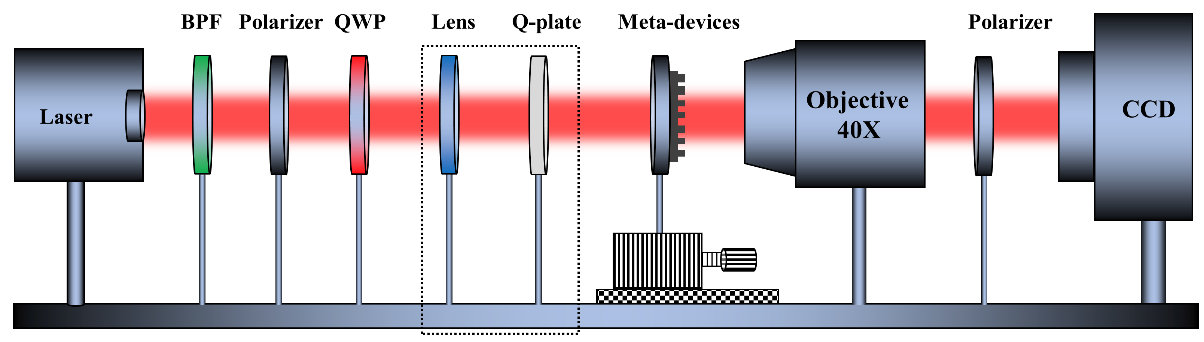


Fig. S6 Characterizing system for measuring the SoPs of incident beams on different HOPSs by using the designed MPPC. BPF: bandpass filter, QWP: quarter waveplate, BS: beam splitter, and CCD: charge-coupled device. The dashed boxes indicate that the lens and q-plate are removable in the certain measuring process.

We utilize the setup shown in Figure S6 to characterize the SoPs of incident beams on different HOPSs. A linear polarizer and QWP pair is used to generate arbitrary SoPs on the 0-order HOPS. For generating arbitrary SoPs on the 1-order and 2-order HOPSs, the linear polarizer and QWP pair along with the commercially available *q*-plates (with *q* values of 0.5 and 1) are utilized. The linear polarizer placed behind the CCD is used to extract the specific electric component.

Here we give the detailed formula derivation process for generating arbitrary SoPs on the 1-order and 2-order HOPSs. The Jones matrix of the *q*-plate can be expressed as

$J_{q}=\left[ \begin{matrix} cos2\alpha& sin2\alpha\\ sin2\alpha& -cos2\alpha\end{matrix} \right]$ (S15)

where $\alpha=q\varphi+\varphi_{0}$, *q* is the topological charge, $\varphi$ is the azimuth angle, $\varphi_{0}$ is the initial phase. If a *x*-polarized incident light (|*X*> = [1, 0]^T^) is passing through the *q*-plate with its polarization direction parallel to the optical axis of *q*-plate, the output polarization field can be expressed as

$E_{out}=\left[ \begin{matrix} cos2\alpha& sin2\alpha\\ sin2\alpha& -cos2\alpha\end{matrix} \right]\left[ \begin{matrix} 1 \\ 0 \end{matrix} \right]=\left[ \begin{matrix} cos(2q\varphi) \\ sin(2q\varphi) \end{matrix} \right]$ (S16)

From Eq. S16, one can see that a radially vector beam is generated (when *q* = 1/2). Similarly, when the incident light is switched to *y*-polarized incident light (|*Y*> = [0, 1]^T^), the output polarization field can be expressed as

$E_{out}=\left[ \begin{matrix} cos2\alpha& sin2\alpha\\ sin2\alpha& -cos2\alpha\end{matrix} \right]\left[ \begin{matrix} 0 \\ 1 \end{matrix} \right]=\left[ \begin{matrix} sin(2q\varphi) \\ -cos(2q\varphi) \end{matrix} \right]$ (S17)

From Eq. S17, one can see that an azimuth vector beam is generated (when *q* = 1/2). Similarly, when the incident light is switched to RCP and LCP incident light (|R> = [1, -i]^T^ and |L> = [1, i]^T^), the output polarization field can be expressed as

$E_{out}=\left[ \begin{matrix} cos2\alpha& sin2\alpha\\ sin2\alpha& -cos2\alpha\end{matrix} \right]\left[ \begin{matrix} 1 \\ -i \end{matrix} \right]=e^{-i2q\varphi}\left[ \begin{matrix} 1 \\ i \end{matrix} \right]$ (S18)

$E_{out}=\left[ \begin{matrix} cos2\alpha& sin2\alpha\\ sin2\alpha& -cos2\alpha\end{matrix} \right]\left[ \begin{matrix} 1 \\ i \end{matrix} \right]=e^{i2q\varphi}\left[ \begin{matrix} 1 \\ -i \end{matrix} \right]$ (S19)

From Eq. S18 and S19, one can see that two circularly polarized vortex beam are generated. Therefore, for a generalized case that the incident light is elliptical polarized (|*E*> = [cos$\chi$, $e^{i\delta}sin\chi$]^T^ ,where $\chi$ and $\delta$ represent the azimuth and ellipticity of the

polarization ellipse, respectively.), the output polarization field can be expressed as

$E_{out}=\left[ \begin{matrix} cos2\alpha& sin2\alpha\\ sin2\alpha& -cos2\alpha\end{matrix} \right]\left[ \begin{matrix} cos\chi\\ e^{i\delta}sin\chi\end{matrix} \right]=\left[ \begin{matrix} cos(2q\varphi)cos\chi+sin(2q\varphi)e^{i\delta}sin\chi\\ sin(2q\varphi)cos\chi-cos(2q\varphi)e^{i\delta}sin\chi\end{matrix} \right]$ (S20)

From Eq. S20, when $\chi$ and $\delta$ take different values, the generated vector beams is able to cover the whole surface of the HOPS.

**6. Resolving the SoPs on the HOPS_0,0_**

**
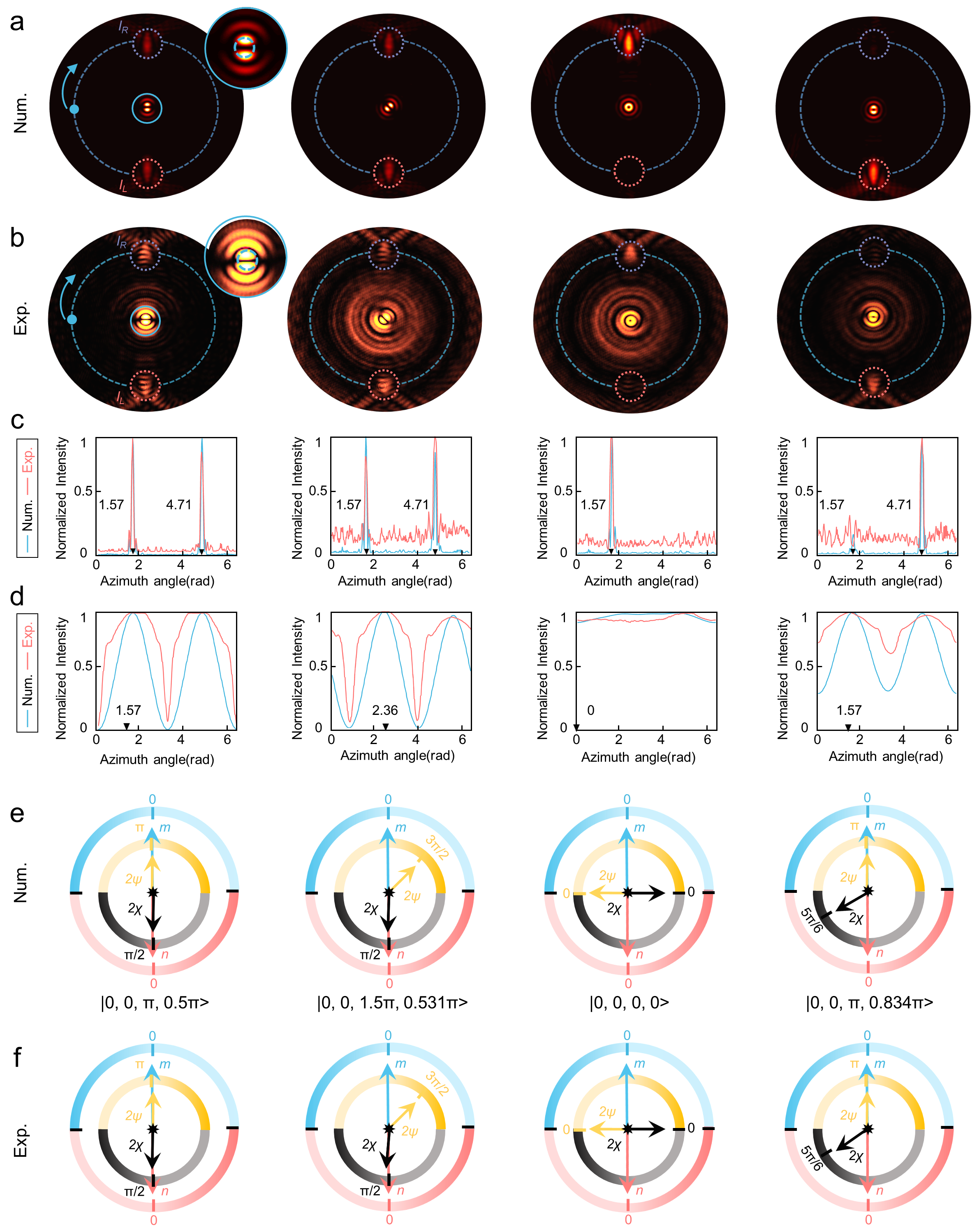
**

Fig. S7 Resolving SoP of beams on the HOPS_0,0_ by using the MPPC. **a** and **b** Simulated and measured intensity (|*E_x_*|^2^) profiles of the MPPC for incident light with four SoPs from left to right: |0, 0, π, π/2>, |0, 0, 3π/2, π/2>, |0, 0, 0, 0>, and |0, 0, 0, 5π/6>. **c**. The normalized intensity distributions along the turquoise dashed ring shown in (**a**) and (**b**). The turquoise and rose red solid lines represent the simulated and measured results, respectively. **d**. The normalized intensity distributions along the turquoise dashed ring shown in the enlarged insets of (**a**) and (**b**). The turquoise and rose red solid lines represent the simulated and measured results, respectively. The black triangle marks in (**c**) and (**d**) represent the azimuth angle of the energy peaks. **e** and **f** Numerically and experimentally retrieved SoPs by the MPPC, where the SoP can be directly readout by four pointers.

**6.1 The calculation of the ellipticity angle**


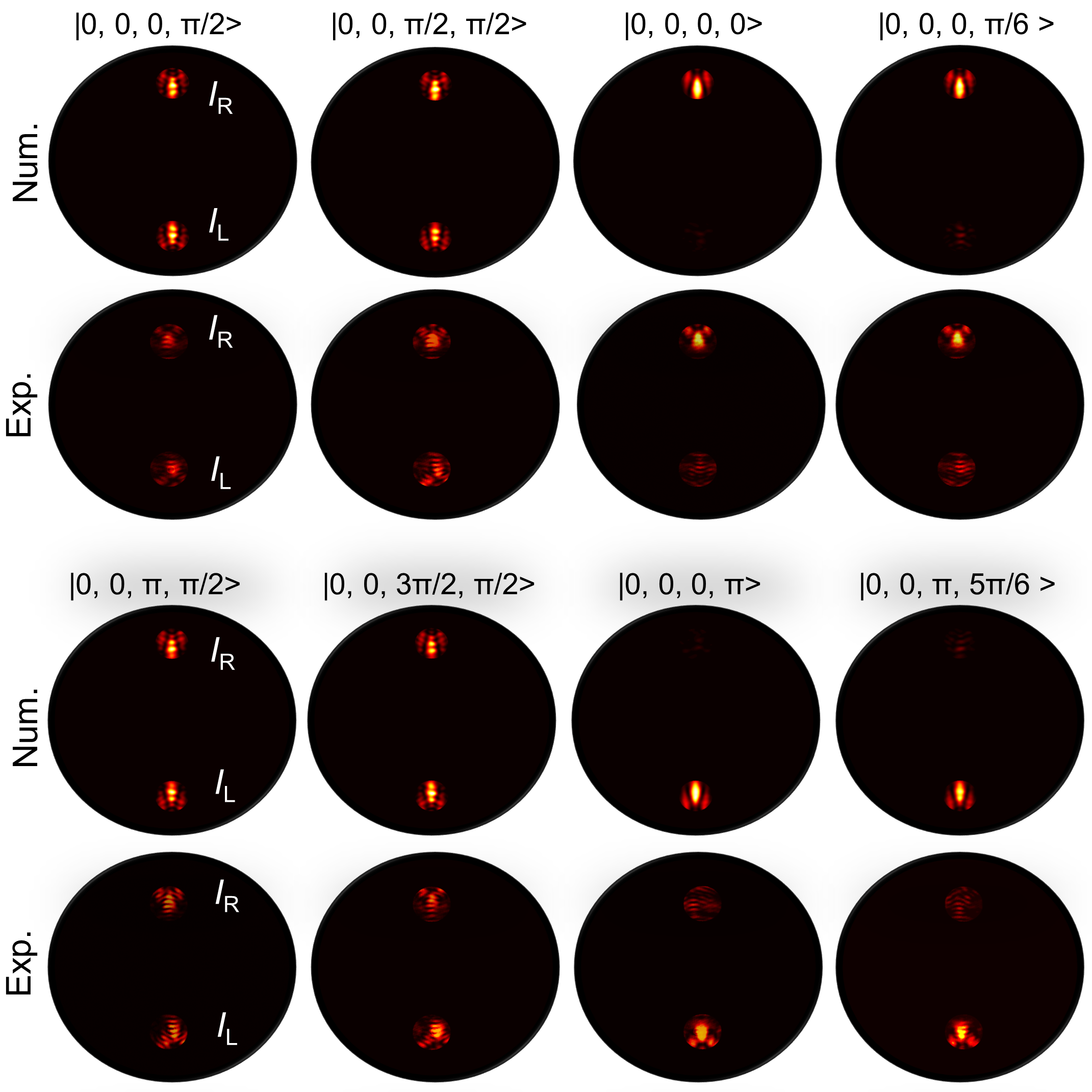


Fig. S8 (a) and (b) Numerical and measured intensity (|*E_x_*|^2^) profiles of the two focal spots for incident light with eight SoPs as |0, 0, 0, π/2>, |0, 0, π/2, π/2>, |0, 0, π, π/2>, |0, 0, 3π/2, π/2>, |0, 0, 0, 0>, |0, 0, 0, π>, |0, 0, 0, π/6> and |0, 0, π, 5π/6>. Here, we just show the intensity profiles inside a circle with radius *r,* with the intensities in the other region are set as zero.

As shown in Fig. 4 in the main text, the two transverse focusing spots also show ellipticity-dependent intensity contrast, from which we can calculate the ellipticity angle. Figure S8 shows the numerical and measured intensity (|*E_x_*|^2^) profiles of the two focal spots for incident light with eight SoPs from left to right of |0, 0, 0, π/2>, |0, 0, π/2, π/2>, |0, 0, π, π/2>, |0, 0, 3π/2, π/2>, |0, 0, 0, 0>, |0, 0, 0, π>, |0, 0, 0, π/6> and |0, 0, π, 5π/6>. Here, we just show the intensity profiles inside a circle with radius *r*, with the intensities in all the other regions are set as zero. Without loss of generality, we set the value of radius *r* according to the definition of focusing efficiency of metalenses [5]. As a result, the radius *r* is set as three times of the focal spot’s FWHM. The intensities on the upper and lower sides of the transverse plane (which corresponds to the RCP and LCP components) are denoted as *I_R_* and *I_L_*, respectively. Here, we just show the intensity profiles inside a circle with radius r, with the intensities in all the other regions are set as zero. Without loss of generality, we set the value of radius *r* according to the definition of focusing efficiency of metalenses [5]. As a result, the radius *r* is set as three times of the focal spot’s FWHM. Then, the Stokes parameter *S*_3_ of the incident beam can be calculated as *S*_3_ *=* (*I_R_ – I_L_*)/(*I_R_ + I_L_*). Hence, the ellipticity angle can be calculated as

$2\chi=\frac{\pi}{2}-\arcsin\left( S_{3} \right),0\leq\chi\leq\frac{\pi}{2}$ (S21)

In Eq. S21, the background intensity is not take into consideration. In the actual calculate process, the intensity *I_L_* for the case of incident RCP SoP (|0, 0, 0, 0>) is regarded as the background intensity, which denoted as *I_b_*. Hence, the Stokes parameter *S*_3_ of the incident beam can be calculated as *S*_3_ *=* (*I_R_ – I_L_*)/(*I_R_ + I_L_*-2*I_b_*).

**6.2 Reconstructed accuracy of the MPPC**

Table 1 shows the numerically and experimentally reconstructed SoPs on the 0-order HOPS. The numerically reconstructed SoPs show well agreement with the original ones, verifying the veracity of our designed MPPC. The experimentally reconstructed SoPs show small discrepancies from the original ones, which can be attributed to the zero-order background, fabrication derivations and measurement errors. Therefore, via a single measurement, the MPPC is able to fully resolve an arbitrary beam on the 0-order HOPS. To visualize the results, we depict the original, simulated and measured SoPs on the 0-order HOPS, as shown in Fig. S9.

**Table 1 Numerically and experimentally reconstructed SoPs on the 0-order HOPS**.

| Original SoP  \|*m*, *n*, 2*ψ*, 2*χ*> | Numerically reconstructed SoP | Numerical Error | Experimentally reconstructed SoP | Experimental Error |
| --- | --- | --- | --- | --- |
| \|0, 0, 0, π/2> | \|0, 0, 0, 0.503π> | 0.002 | \|0, 0, 0, 0.488π> | 0.009 |
| \|0, 0, π/2, π/2> | \|0, 0, π/2, 0.468π> | 0.025 | \|0, 0, π/2, 0.470π> | 0.024 |
| \|0, 0, π, π/2> | \|0, 0, π, 0.5π> | 0.000 | \|0, 0, 1.006π, 0.504π> | 0.008 |
| \|0, 0, 3π/2, π/2> | \|0, 0, 3π/2, 0.531π> | 0.049 | \|0, 0, 1.521π, 0.558π> | 0.062 |
| \|0, 0, 0, 0> | \|0, 0, 0, 0> | 0.000 | \|0, 0, 0, 0> | 0.000 |
| \|0, 0, 0, π> | \|0, 0, 0, 0.993π> | 0.006 | \|0, 0, 0, 0.961π> | 0.031 |
| \|0, 0, 0, π/6> | \|0, 0, 0, 0.156π> | 0.008 | \|0, 0, 0, 0.194π > | 0.021 |
| \|0, 0, π, 5π/6> | \|0, 0, π, 0.834π> | 0.001 | \|0, 0, π, 0.795π> | 0.030 |


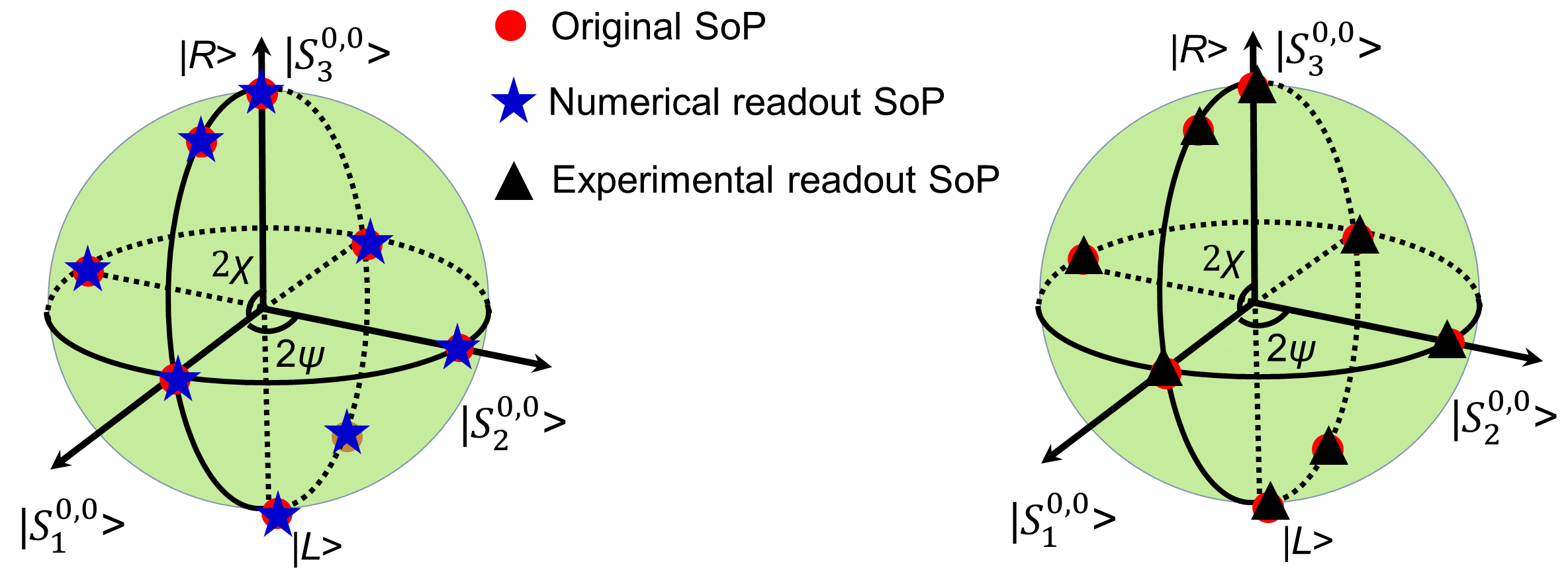


Fig. S9 The original (red points), numerically reconstructed (blue asterisks) and experimentally reconstructed (black triangles) SoPs represented on the 0-order HOPS.

**7. Detecting the SoPs on the 1-order and 2-order HOPSs**


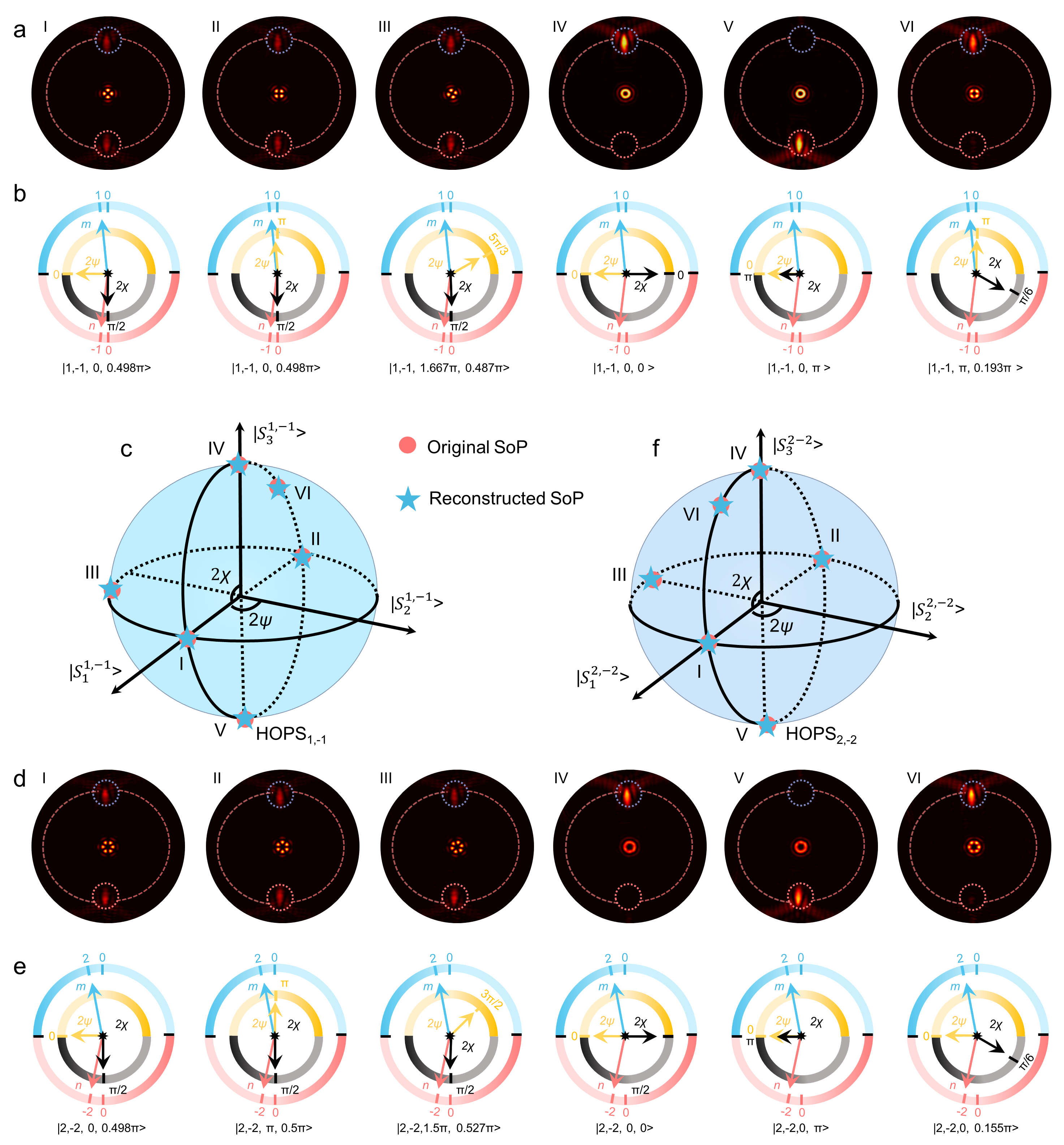


Fig.S10 Numerically resolving SoPs on the 1-order and 2-order HOPSs. (a) Calculated electric field component (|*E_x_*|^2^) profiles of the MPPC for incident light with six SoPs from left to right: |1, -1, 0, π/2>, |1, -1, π, π/2>, |1, -1, 5π/3, π/2>, |1, -1, 0, 0>, |1, -1, 0, π>, and |1, -1, π, π/6>. (b) Numerically retrieved SoPs by the MPPC, where the SoPs can be directly readout by four pointers. (c) Original (red points) and reconstructed (blue asterisks) SoPs represented on the 1-order HOPS. (d) Calculated intensity (|*E_x_*|^2^) profiles of the MPPC for incident light with six SoPs from left to right: |2, -2, 0, π/2>, |2, -2, π, π/2>, |2, -2, 3π/2, π/2>, |2, -2, 0, 0>, |2, -2, 0, π>, and |2, -2, 0, π/6>. (e) Numerically retrieved SoPs by the MPPC, where the SoPs can be directly readout by four pointers. (f) Original (red points) and reconstructed (blue asterisks) SoPs represented on the 2-order HOPS.

Here, we numerically demonstrate fully resolving the SoPs on the 1-order HOPS_+1, -1_ and 2-order HOPS_+2, -2_ by using the MPPC, as shown in Fig. S10. Fig. S10a shows the calculated intensity (|*E_x_*|^2^) profiles of the MPPC under different incident beams on the 1-order HOPS. The incident beams with six SoPs |1, -1, 0, π/2>, |1, -1, π, π/2>, |1, -1, 5π/3, π/2>, |1, -1, 0, 0>, |1, -1, 0, π>, and |1, -1, π, π/6> located on the surface of the 1-order HOPS are chose as typical examples. By analyzing the generated intensity patterns, we can retrieve the four parameters |*m*, *n*, *2ψ,* 2*χ*>. As shown in Fig. S10b, the six SoPs can be readout by the MPPC with four independent pointers. Figure S10c shows the representation of the original and reconstructed SoPs on the 1-order HOPS. It is obvious that the numerically reconstructed SoPs show well agreement with the original ones. Figure S10d shows the calculated intensity (|*E_x_*|^2^) profiles of the MPPC under different incident beams on the 2-order HOPS. The incident beams with six SoPs |2, -2, 0, π/2>, |2, -2, π, π/2>, |2, -2, 3π/2, π/2>, |2, -2, 0, 0>, |2, -2, 0, π>, and |2, -2, 0, π/6> located on the surface of the 2-order HOPS are chose as typical examples. Similarly, the four parameters |*m*, *n*, *2ψ,* 2*χ*> of an arbitrary beam on the 2-order HOPS can be retrieved by analyzing the generated intensity patterns. Figure S10f shows the representation of the original and reconstructed SoPs on the 2-order HOPS, from which we can see that the numerically reconstructed SoPs show well agreement with the original ones.


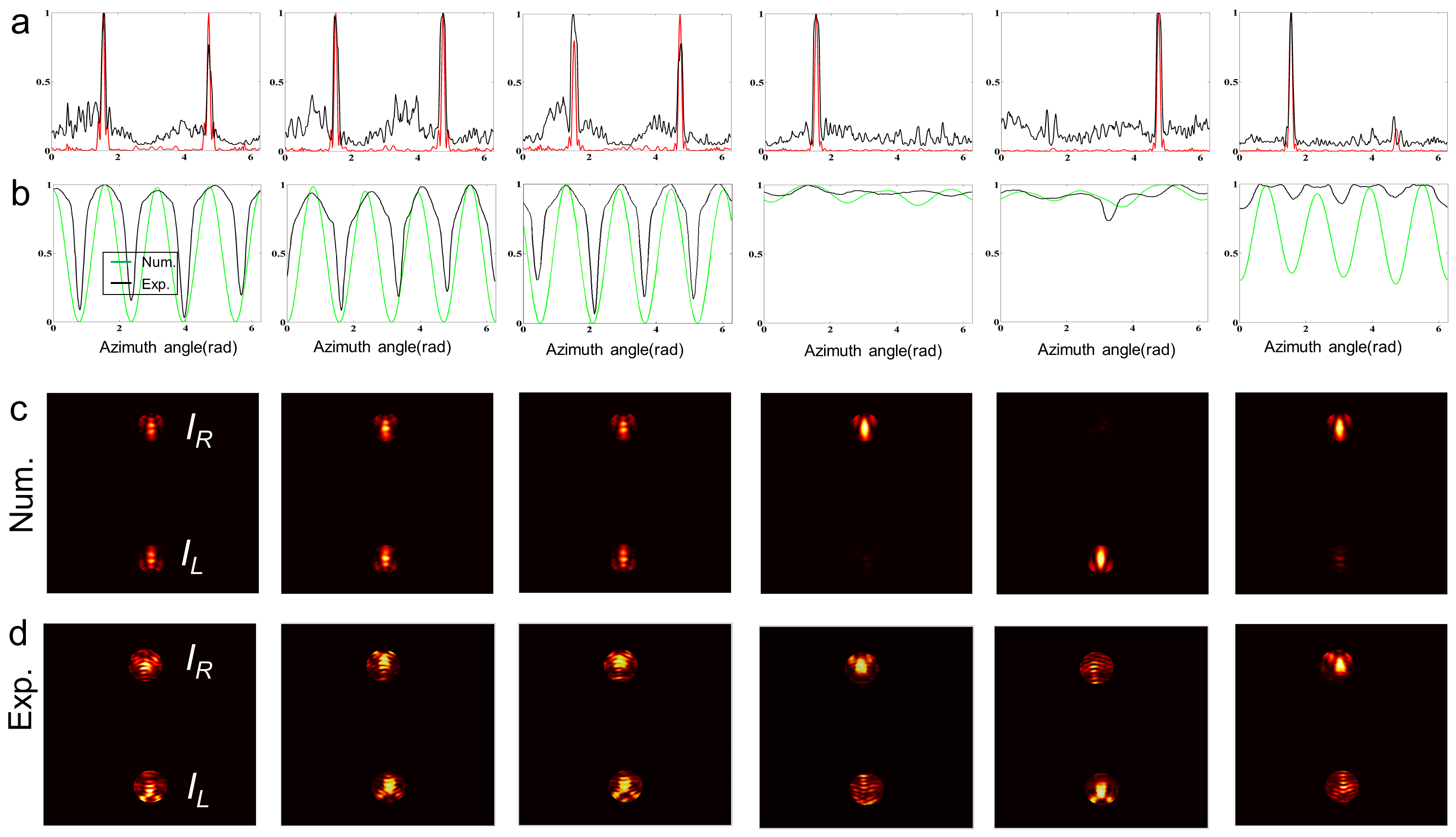


Fig.S11 (a) The normalized intensity distributions along the turquoise dashed ring shown in figure 5**a** in the main text. The red and black solid lines represent the simulated and measured results, respectively. (b) The normalized intensity distributions along the inner ring in figure 5**a** in the main text. The green and black solid lines represent the simulated and measured results, respectively. (c) and (d) Numerical and measured intensity profiles of the two focal spots for incident light with six SoPs as |1, -1, 0, π/2>, |1, -1, π, π/2>, |1, -1, 5π/3, π/2>, |1, -1, 0, 0>, |1, -1, 0, π>, and |1, -1, π, π/6>. Here, we just show the intensity profiles inside a circle with radius *r*, with the intensities in the other region are set as zero.


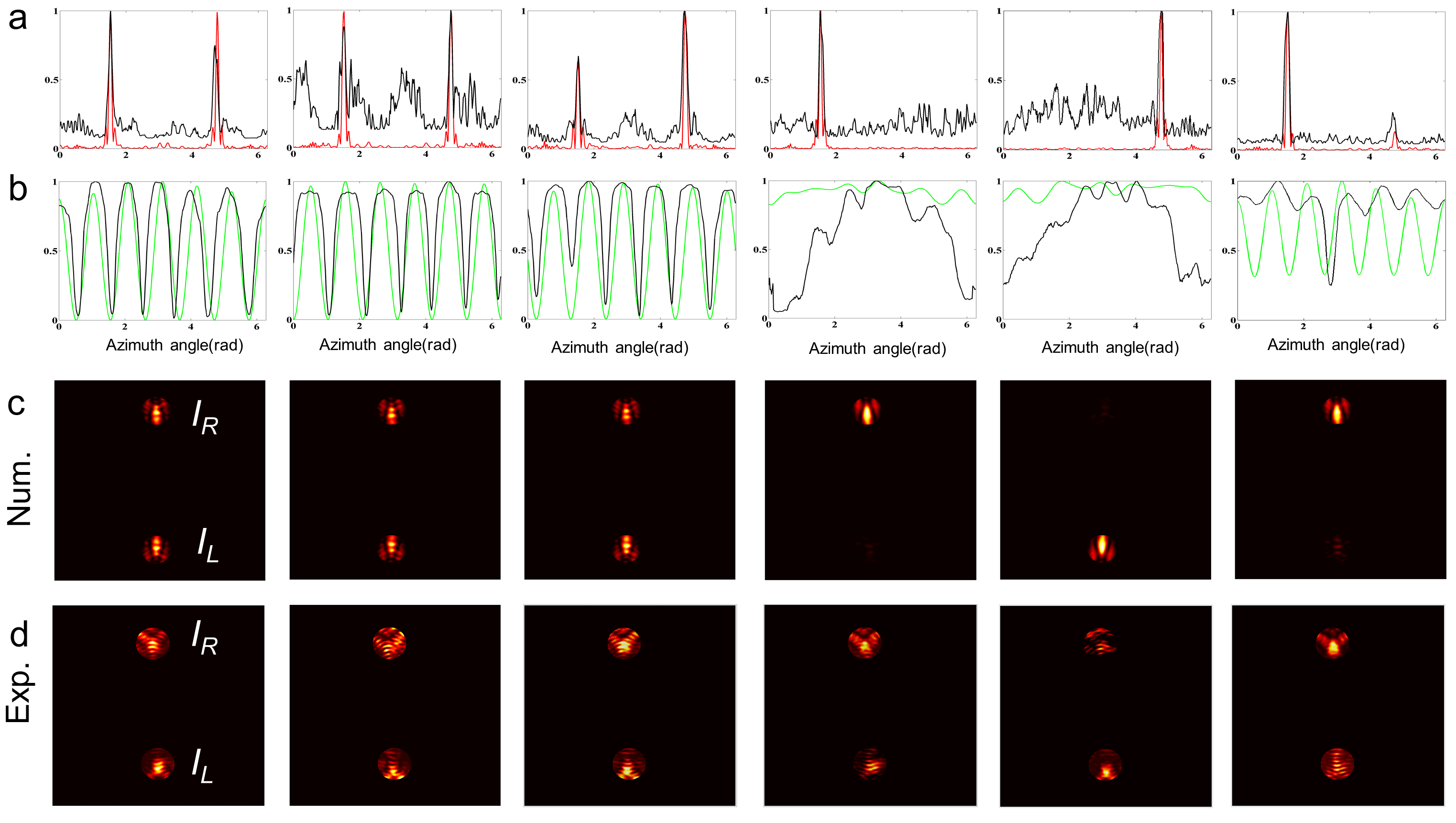


Fig.S12 (a) The normalized intensity distributions along the turquoise dashed ring shown in figure 5**d** in the main text. The red and black solid lines represent the simulated and measured results, respectively. (b) The normalized intensity distributions along the inner ring in figure 5**d** in the main text. The green and black solid lines represent the simulated and measured results, respectively. (c) and (d) Numerical and measured intensity profiles of the two focal spots for incident light with six SoPs as |2, -2, 0, π/2>, |2, -2, π, π/2>, |2, -2, 3π/2, π/2>, |2, -2, 0, 0>, |2, -2, 0, π>, and |2, -2, 0, π/6>. Here, we just show the intensity profiles inside a circle with radius *r*, with the intensities in the other region are set as zero.

Table 2 and table 3 show the numerically and experimentally reconstructed SoPs on the 1-order HOPS and 2-order HOPS. For both cases, the numerically reconstructed SoPs show well agreement with the original ones, verifying the veracity of our designed MPPC. The experimentally reconstructed SoPs show small discrepancies from the original ones, which can be attributed to the zero-order background, fabrication derivations and measurement errors. Therefore, via a single measurement, the MPPC is able to fully resolve an arbitrary beam on the 1-order HOPS and 2-order HOPS.

**Table 2 Numerically and experimentally reconstructed SoPs on the 1-order HOPS**.

| Original SoP  \|*m*, *n*, 2*ψ*, 2*χ*> | Numerically reconstructed SoP | Numerical Error | Experimentally reconstructed SoP | Experimental Error |
| --- | --- | --- | --- | --- |
| \|1, -1, 0, π/2> | \|1,-1,0,0.498π> | 0.002 | \|1,-1,0.043π,0.533π> | 0.059 |
| \|1, -1, $\pi$, π/2> | \|1,-1, $\pi$,$0.498\pi$> | 0.002 | \|1,-1, 0.946π,0.562π> | 0.091 |
| \|1, -1, 5π/3, π/2> | \|1,-1, 5π/3,0.487π> | 0.010 | \|1,-1, 5π/3,0.512π> | 0.009 |
| \|1, -1, 0, 0> | \|1,-1,0,0> | 0.000 | \|1,-1,0,0> | 0.000 |
| \|1, -1, 0, π> | \|1,-1,0,π> | 0.000 | \|1,-1,0,0.889π> | 0.079 |
| \|1, -1, π, π/6> | \|1,-1, π,0.193π> | 0.021 | \|1,-1, $\pi$,0.2$11\pi$> | 0.035 |

**Table 3 Numerically and experimentally reconstructed SoPs on the 2-order HOPS**.

| Original SoP  \|*m*, *n*, 2*ψ*, 2*χ*> | Numerically reconstructed SoP | Numerical Error | Experimentally reconstructed SoP | Experimental Error |
| --- | --- | --- | --- | --- |
| \|2, -2, 0, π/2> | \|2,-2,0,0.498π> | 0.002 | \|2,-2,0.057π,0.559π> | 0.091 |
| \|2, -2, 0, π/2> | \|2,-2, π,0.5π> | 0.000 | \|2,-2, 0.964π,0.549π> | 0.067 |
| \|2, -2, 3π/2, π/2> | \|2,-2, 3π/2,0.527π> | 0.021 | \|2,-2, 1.763π,0.393π> | 0.291 |
| \|2, -2, 0, 0> | \|2,-2,0,0> | 0.000 | \|2,-2,0,0> | 0.000 |
| \|2, -2, 0, π> | \|2,-2,0,π> | 0.000 | \|2,-2,0,0.856π> | 0.113 |
| \|2, -2, 0, π/6> | \|2,-2,0,0.155π> | 0.009 | \|2,-2, 0.083π,0.218π> | 0.106 |

**8. Numerically reconstructed the SoPs on the hybrid and higher orderHOPS**

To further validate the functionality of the proposed MPPC, we consider fully resolving the SoPs on the hybrid-order HOPS_2,-1_ by using the MPPC, as shown in Fig. S13. Here, we just show the numerical results for the fact that there are no commercial element for generating SoPs on the hybrid-order HOPS. As a typical example, we choose the hybrid-order HOPS of HOPS_2,-1_, indicating that the optical singularities are *m* = 2 and *n* = -1. Fig. S13a shows the calculated intensity (|*E_x_*|^2^) profiles of the MPPC under different incident beams on the HOPS_2,-1_. The incident beams with four SoPs |2, -1, 0, π/2>, |2, -1, π/2, π/2>, |2, -1, 0, π>, and |2, -1, π, -π/12> located on the surface of the HOPS_2,-1_ are chose as typical examples. By analyzing the generated intensity patterns, we can retrieve the four parameters |*m*, *n*, *2ψ,* 2*χ*>.

Figure S13b depicts the intensity distributions along the red dashed rings (take the leftmost of the ring as the starting point and go anti-clockwise) in Fig. S13a. The focusing spots that located on the upper and lower part of the transverse plane indicate that the value of SAM are -1 and +1, respectively. The orientation angle of the focusing spot determines the topological charge of the two CP components. By analyzing the generated intensity patterns, we can retrieve the four parameters |*m*, *n*, *2ψ,* 2*χ*>. As shown in Fig. S13d, the four SoPs can be readout by the MPPC with four independent pointers.


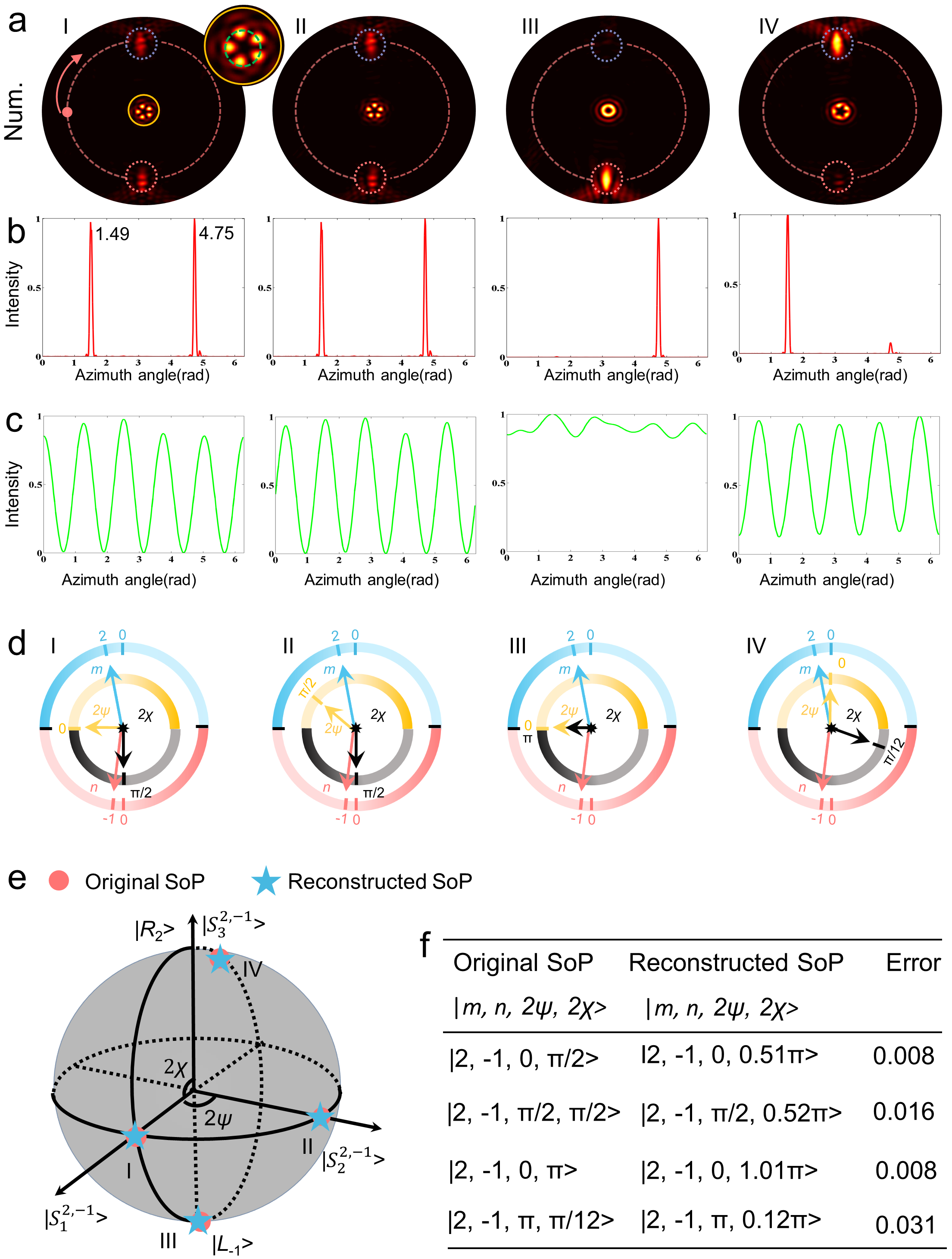


Fig.S13 Numerically resolving SoPs on the hybrid-order HOPS expressed as HOPS_2,-1_. (a) Calculated intensity (|*E_x_*|^2^) profiles of the MPPC for incident light with four SoPs from left to right: |2, -1, 0, π/2>, |2, -1, π/2, π/2>, |2, -1, 0, π>, and |2, -1, π, -π/12>. (b) The intensity distributions along the rose red dashed ring shown in (a). (c) The intensity distributions along the green dashed ring shown in (a). (d) Numerically retrieved SoPs by the MPPC, where the SoPs can be directly readout by four pointers. (e) Original (red points) and reconstructed (blue asterisks) SoPs represented on the hybrid-order HOPS. (f) Numerical errors between the original and reconstructed SoPs.


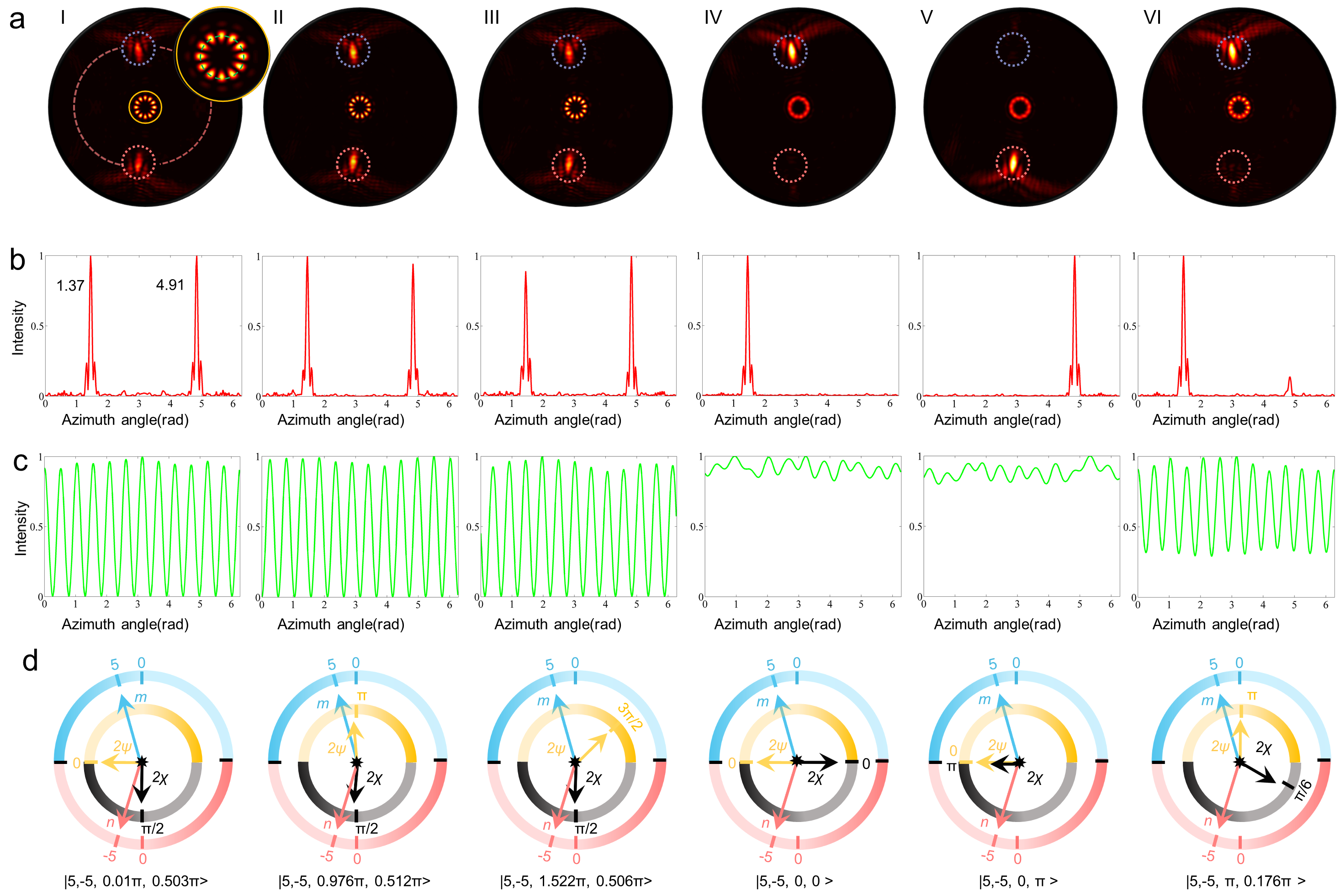


**Fig. S14** Theoretically resolving SoPs on the 5th order HOPS expressed as HOPS_5,-5_. (a) Calculated intensity (|*E_x_*|^2^) profiles of the MPPC for incident light with six SoPs from left to right: |5, -5, 0, π/2>, |5, -5, π, π/2>, |5, -5, 3π/2, π/2>, |5, -5, 0, 0>, |5, -5, 0, π>, and |5, -5, π, π/6>. (b) The intensity distributions along the rose red dashed ring shown in (a). (c) The intensity distributions along the green dashed ring shown in (a). (d) Numerically retrieved SoPs by the MPPC, where the SoPs can be directly readout by four pointers.

To further underscore the robustness and versatility of the MPPC, we have theoretically demonstrated fully resolving the SoPs on the 5th order HOPS by using the MPPC, as depicted in Fig. S14. Without loss of generality, we picked up six different vector states on the HOPS_5,-5_ to prove our designed MPPC. The six SoPs are |5, -5, 0, π/2>, |5, -5, π, π/2>, |5, -5, 3π/2, π/2>, |5, -5, 0, 0>, |5, -5, 0, π>, and |5, -5, π, π/6>. Fig. S14a shows the calculated intensity (|*E_x_*|^2^) profiles of the MPPC under different incident beams on the HOPS_5,-5_. Figure S14b depicts the intensity distributions along the red dashed rings (take the leftmost of the ring as the starting point and go anti-clockwise) in Fig. S14a. The focusing spots that located on the upper and lower part of the transverse plane indicate that the topological charges of the two CP vortex beams are *m* = 5 and *n* = -5, respectively. By analyzing the generated intensity patterns, we can retrieve the four parameters |*m*, *n*, *2ψ,* 2*χ*>. As shown in Fig. S13d, the four SoPs can be readout by the MPPC with four independent pointers.

**9. Reconstructed the SoPs on an arbitrary HOPS**

According to the design principle of the MPPC in the main text, the intrinsic optical singularities of MPPC’s inner part are |*σ* = -1, *m* = +1> and |*σ* = +1, *n* = -1>, namely, RCP mode with topological charge +1 and LCP mode with topological charge +1. Hence, depending on the superposition principle of angular momentum (AM), such a meta-device is able to resolving SoPs on an arbitrary HOPS except the one with optical singularities of |*σ* = -1, *m* = -1> and |*σ* = +1, *n* = +1>. Figure S15a shows the calculated intensity (|*E_x_*|^2^) profiles of the MPPC for incident light on the HOPS_-1,+1_, with five SoPs from left to right as |-1, +1, 0, π/2>, |-1, +1, π/2, π/2>, |-1, +1, 0, 0>, |-1, +1, 0, π>, and |-1, +1, 0, π/6>. It is obvious that the intensity profiles in the central area exhibits focal spots for all these cases. The underlying physics is that the total value of AM is zero according to the superposition principle of AM. As a result, for incident beams on the HOPS_-1,+1_, the polarization azimuth angle *ψ* cannot be determined.


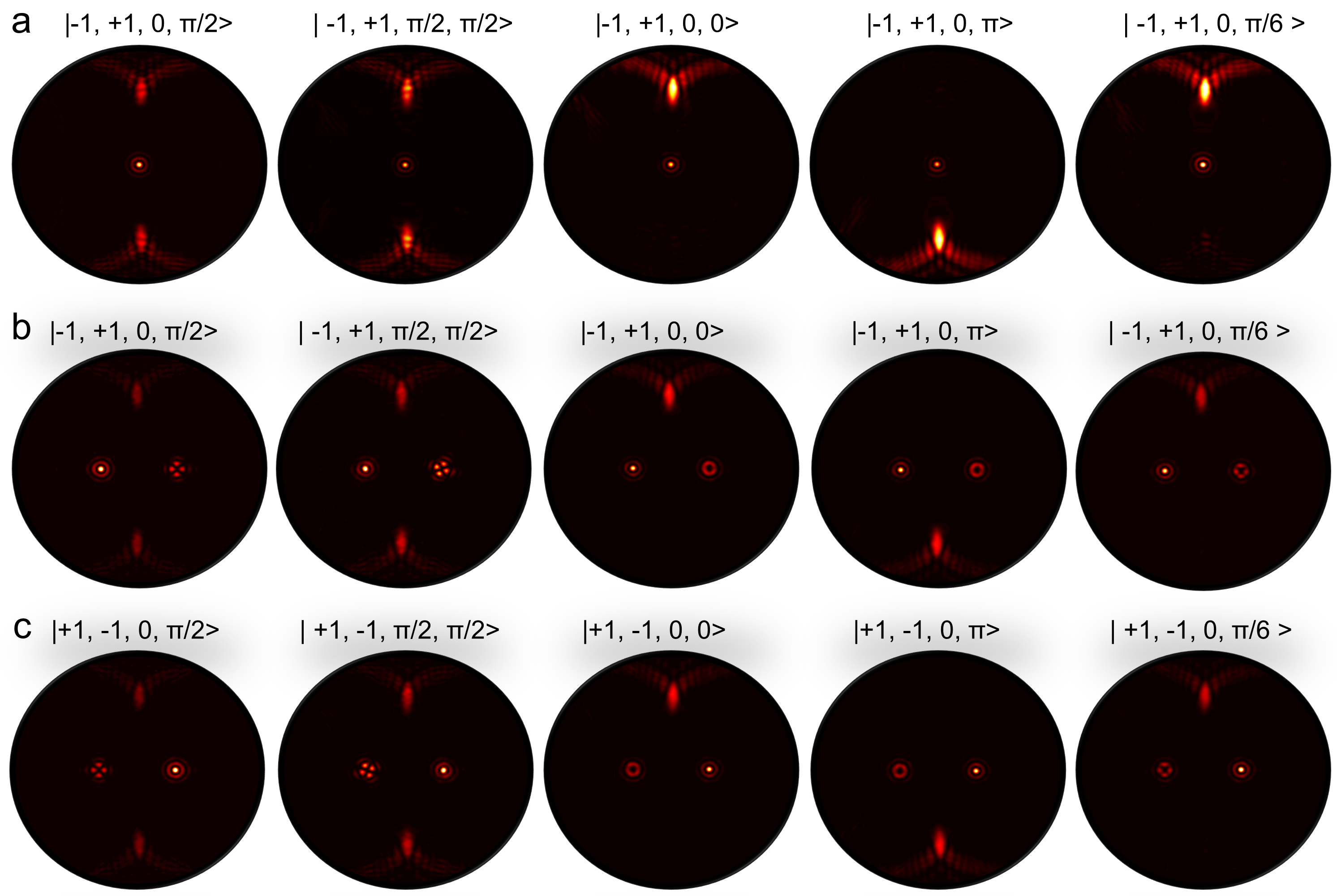


Fig.S15 (a) Calculated intensity (|*E_x_*|^2^) profiles of the MPPC for incident light on the HOPS_-1,+1_, with five SoPs from left to right as |-1, +1, 0, π/2>, |-1, +1, π/2, π/2>, |-1, +1, 0, 0>, |-1, +1, 0, π>, and |-1, +1, 0, π/6>. (b) Calculated intensity profiles of the modified MPPC for incident light on the HOPS_-1,+1_, with five SoPs from left to right as |-1, +1, 0, π/2>, |-1, +1, π/2, π/2>, |-1, +1, 0, 0>, |-1, +1, 0, π>, and |-1, +1, 0, π/6>. (c) Calculated intensity profiles of the modified MPPC for incident light on the HOPS_+1,-1_, with five SoPs from left to right as |+1, -1, 0, π/2>, |+1, -1, π/2, π/2>, |+1, -1, 0, 0>, |+1, -1, 0, π>, and |+1, -1, 0, π/6>.

To solve this issue, we can innovate the design of the MPPC with the identical design strategy. The modified MPPC has two inner parts, which own intrinsic optical singularities of |*σ* = -1, *m* = +1>, |*σ* = +1, *n* = -1> and |*σ* = -1, *m* = -1>,|*σ* = +1, *n* = +1>, respectively. Figure S15b shows the calculated intensity (|*E_x_*|^2^) profiles of the MPPC for incident light on the HOPS_-1,+1_, with five SoPs from left to right as |-1, +1, 0, π/2>, |-1, +1, π/2, π/2>, |-1, +1, 0, 0>, |-1, +1, 0, π>, and |-1, +1, 0, π/6>. According to the superposition principle of AM, the intensity profiles in the left central area exhibit focal spots for all these cases, while the intensity profiles in the right central area exhibit petal-shaped pattern. Therefore, according to the discussion in the main text, the modified MPPC is able to fully resolving the SoPs on the HOPS_-1,+1_. Moreover, the SoPs on the HOPS_+1,-1_ is also take into consideration. Figure S15c shows the calculated intensity (|*E_x_*|^2^) profiles of the MPPC for incident light on the HOPS_+1,-1_, with five SoPs from left to right as |+1, -1, 0, π/2>, |+1, -1, π/2, π/2>, |+1, -1, 0, 0>, |+1, -1, 0, π>, and |+1, -1, 0, π/6>. It is observed that the intensity profiles in the right central area exhibit focal spots for all these cases, while the intensity profiles in the left central area exhibit petal-shaped pattern. Therefore, the design principle of the MPPC is general, which can be used to resolve SoPs of beam on an arbitrary HOPS.

**10. Free-space optical communication using vector beams**

Currently, vector beams have raised a range of important applications in various fields such as optical communication, display technology and encryption methods, particle manipulation techniques, laser direct writing, high-dimensional quantum information processing. Here we rehearse a vector beam optical communication system that enables the utilization of vector beams (VBs) positioned at arbitrary locations on the HOPS, relying on the pioneering work that focus on VB communication [6]. Without loss of generality, we choose six VBs on the 1th order HOPS as typical example. The corresponding SoPs are |1, -1, 0, π/2>, |1, -1, π, π/2>, |1, -1, 5π/3, π/2>, |1, -1, 0, 0>, |1, -1, 0, π>, and |1, -1, π, π/6>. The schematic of the VB optical communication system is shown in Fig. S16a. The incoming laser beam is split into N copies, each passing through a VB generation module that consists of a polarizer, a quarter-wave plate and a *q*-plate. Here, as a proof of concept, we just shows six channels, which are marked with roman numerals from I to VI. Then, the generated VBs are calibrated by using our designed MPPC, leading to specific patterns for each VB. To visualize the generated VBs, the six VBs are represented with black points on the HOPS_1,-1_, as depicted in Fig. S16b. The VBs are then propagate through a free-space optical link with turbulence cell. At the receiver end, the received vector modes are passing through a decoding channel designed for the identification of the vector modes. Noting that our designed MPPC is also utilized as the decoding device, which capable of transforming the vector modes into specific patterns. Finally, the structural similarity index measure (SSIM) algorithm is used to calculate the similarity index of the obtained patterns of vector beam in the calibration and decoding processes [7]. The detected signals of six decoding channels for six input modes are shown in Fig. S16c. All six decoded signals (the maximum value of the similarity index corresponds to the value of “1”, otherwise the value is “0”) are then compared to determine the final detected information level (VB modes).

The proposed vector beam (VB) optical communication system can be used for information processing. Since the spatial polarization profile is determined by the relative phase (and amplitude) of the two orthogonal vortex circular polarization components, the information encoded in the spatial polarization profiles is essentially encoded as the difference of the spatially-varying phase profiles between the two polarization components. Thus, analogously to the well-known differential phase shift keying (DPSK) protocol in which the information is encoded in the relative phase between neighboring pulses in the time domain, we can also call our protocol spatial polarization differential phase shift keying (SPDPSK), indicating that the information is encoded in the relative phase between the two polarization components of a vector beam. Since the information is encoded in the spatial polarization profile rather than the complex-field profile of the beam, it is better conserved under turbulent conditions [8]. In theory, the spatial polarization profiles on the HOPSs are infinite, indicating the number of information levels that can be encoded is infinitely large.


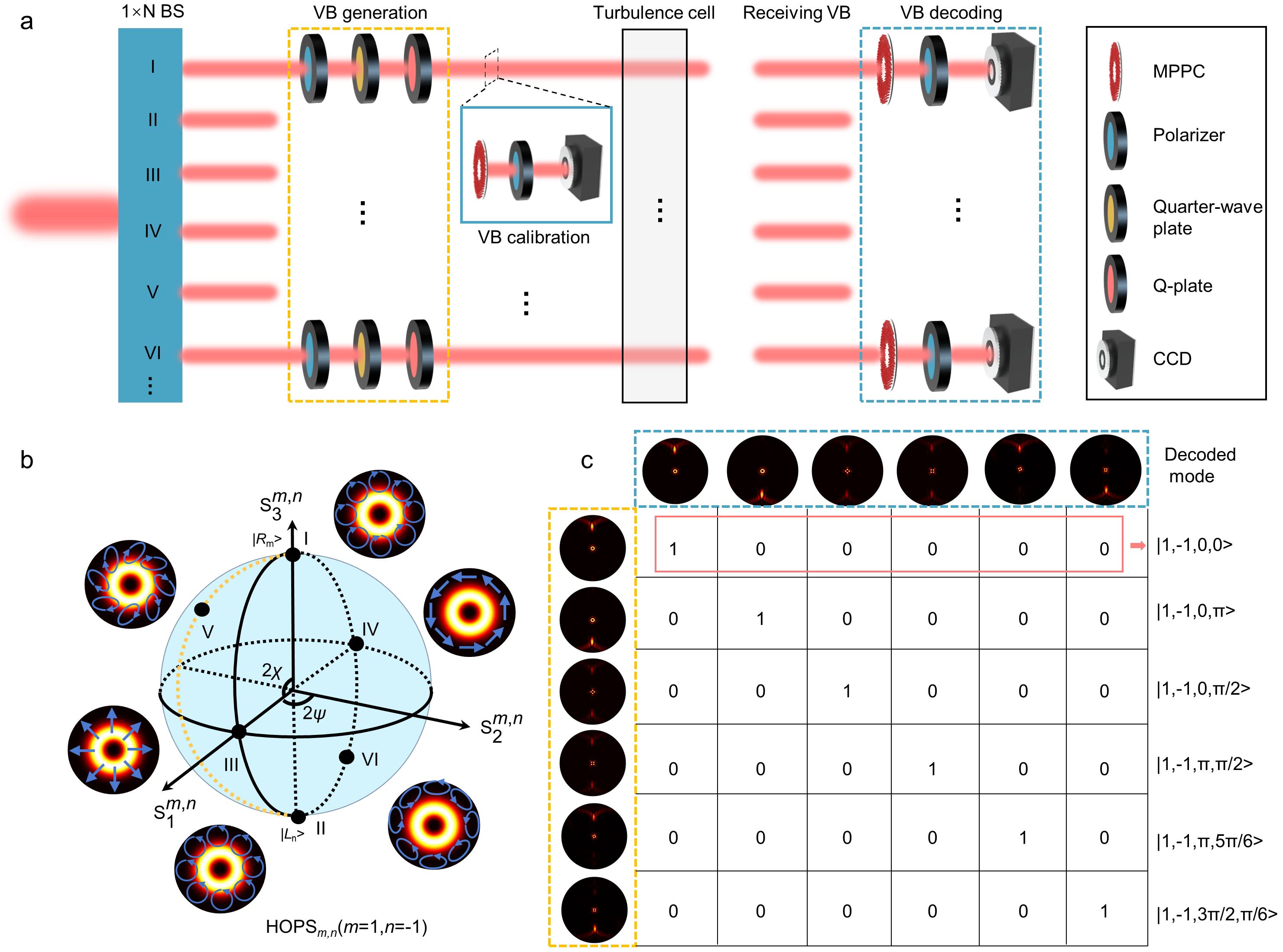


Fig.S16 The schematic of the vector beam (VB) optical communication system. (a) The communication system including a VB generation module, a controllable turbulence cell, and a signal detection module. We choose six vector beams on the 1th order HOPS as typical example. The polarizer, quarter-wave plate and q-plate are cascaded for generating the required VBs. (b) The selected six VBs are represented with black points on the HOPS_1,-1_. The six VBs are generated by the six channels, which are marked with roman numerals from I to VI. (c) The principle of vector mode detection. The blue dashed box shows the transformed patterns of the calibrated VBs by using our designed MPPC. The yellow dashed box shows the transformed patterns of the decoded vector beams by using our designed MPPC. The table shows the detected signals of six decoding channels for six input modes, and the final decoded information are at the right. BS: beam splitter, MPPC: metasurface photonic polarization clock, CCD: charge coupled device.

**References**

[1] G. Milione, H. I. Sztul, D. A. Nolan, et al. “Higher-order Poincaré sphere, Stokes parameters, and the angular momentum of light,” Phys. Rev. Lett. 107, 053601 (2011).

[2] D. Naidoo, F. S. Roux, A. Dudley, et al. “Controlled generation of higher-order Poincaré sphere beams from a laser,” Nat. Photonics, 10, 327–332 (2016).

[3] R. Sahu, S. Chaudhary, K. Khare, et al. “Angular lens,” Optics Express, 26(7): 8709-8718 (2018).

[4] Y. Guo, S. Zhang, M. Pu, et al. “Spin-decoupled metasurface for simultaneous detection of spin and orbital angular momenta via momentum transformation,” Light: Science & Applications, 2021, 10(1): 1-12.

[5] A. Arbabi, Y. Horie, A. J. Ball, et al. “Subwavelength-thick lenses with high numerical apertures and large efficiency based on high-contrast transmitarrays,” Nature Communications, 2015, 6(1): 7069.

[6] Z. Zhu, M. Janasik, A. Fyffe, et al. “Compensation-free high-dimensional free-space optical communication using turbulence-resilient vector beams,” Nature communications, 2021, 12(1): 1666.

[7] Chen M J, Bovik A C. “Fast structural similarity index algorithm,” Journal of Real-Time Image Processing, 2011, 6: 281-287.

[8] Nape I, Singh K, Klug A, et al. “Revealing the invariance of vectorial structured light in complex media,” Nature Photonics, 2022, 16(7): 538-546.
